# A Nanosized Codelivery System Based on Intracellular Stimuli-Triggered Dual-Drug Release for Multilevel Chemotherapy Amplification in Drug-Resistant Breast Cancer

**DOI:** 10.3390/pharmaceutics14020422

**Published:** 2022-02-15

**Authors:** Yufan Guo, Shuo Liu, Fazhen Luo, Dongyun Tang, Tianshu Yang, Xiuru Yang, Yan Xie

**Affiliations:** 1 Research Center for Health and Nutrition, School of Public Health, Shanghai University of Traditional Chinese Medicine, Shanghai 201203, China; guo_yufan@shutcm.edu.cn (Y.G.); shuoliu@medicilon.com.cn (S.L.); lfz1042535664@shutcm.edu.cn (F.L.); 15990360485@shutcm.edu.cn (D.T.); shuyang0122@shutcm.edu.cn (T.Y.); 18263825726@shutcm.edu.cn (X.Y.); 2 Pharmacy Department, Shanghai TCM-Integrated Hospital, Shanghai University of Traditional Chinese Medicine, Shanghai 200082, China; 3 Pharmacy Department, Xiangshan Hospital of Traditional Chinese Medicine, Shanghai 200020, China

**Keywords:** MDR cancer therapy, codelivery system, hybrid polymeric nanoparticles, stimuli-responsiveness, chemotherapy amplification

## Abstract

Lacking nano-systems for precisely codelivering the chemotherapeutics paclitaxel (PTX) and the natural P-glycoprotein (P-gp) inhibitor, quercetin (QU), into cancer cells and controlling their intracellular release extremely decreased the anticancer effects in multidrug resistant (MDR) tumors. To overcome this hurdle, we constructed hybrid polymeric nanoparticles (PNPs) which consist of redox-sensitive PTX/polyethyleneimine-tocopherol hydrogen succinate-dithioglycollic acid PNPs and pH-sensitive hyaluronic acid-QU conjugates. The obtained hybrid PNPs can be internalized into drug-resistant breast cancer cells by the hyaluronic acid/CD44-mediated endocytosis pathway and escape from the lysosome through the “proton sponge effect”. Under the trigger of intracellular stimuli, the nanoplatform used the pH/glutathione dual-sensitive disassembly to release QU and PTX. The PTX diffused into microtubules to induce tumor cell apoptosis, while QU promoted PTX retention by down-regulating P-gp expression. Moreover, tocopherol hydrogen succinate and QU disturbed mitochondrial functions by generating excessive reactive oxygen species, decreasing the mitochondrial membrane potential, and releasing cytochrome c into the cytosol which consequently achieved intracellular multilevel chemotherapy amplification in MDR cancers. Importantly, the PNPs substantially suppressed tumors growth with an average volume 2.54-fold lower than that of the control group in the MCF-7/ADR tumor-bearing nude mice model. These presented PNPs would provide a valuable reference for the coadministration of natural compounds and anticarcinogens for satisfactory combination therapy in MDR cancers.

## 1. Introduction

As the mainstay of oncological treatments, chemotherapy has received increasing attention over the years with the promise to reduce the morbidity and mortality of breast cancer by playing an indispensable role in providing superior therapeutic effects [1,2]. However, sustained (dose/time-dependent) administration of chemotherapeutic agents decidedly leads to the emergence of tumor multidrug resistance (MDR), i.e., cancer cells simultaneously resistant to a broad spectrum of structure/function-nonidentical chemotherapeutics [3,4]. In fact, over 90% of chemotherapy failures amidst patients with malignant tumors are related to MDR, resulting in unfavorable prognosis, recrudescent disease, and a decreased subsistence rate [5,6]. Recently, combination therapy of chemo-drugs with P-glycoprotein (P-gp)-targeted small-molecule inhibitors has been considered an advantageous approach in MDR reversal as numerous anticancer agents belong to substrates of P-gp, which is an overexpressed drug-efflux transporter, especially in malignant cells [7]. Unfortunately, although the present three generations of P-gp suppressants, including verapamil, biricodar, and elacridar, can restore the efficacy of chemotherapeutic drugs in vitro and in vivo, they seldom have been approved for clinical use due to some deficiencies, such as improper interactions with therapeutic agents and intolerable systemic adverse effects [8,9,10]. Hence, identifying a more efficacious and less toxic P-gp inhibitor is needed to achieve better combination treatments.

Quercetin (QU, 3,3′,4′,5,7-pentahydroxyflavone), a bioactive polyphenolic flavonoid ingredient that exists abundantly in onions, berries, apples, nuts, and tea, is also accepted as a dietary supplement to add nutraceuticals for disease prevention due to its bioactivities, such as antioxidation, anti-inflammation, and immunomodulation, etc. [11,12,13]. Furthermore, no significant abnormalities in blood parameters, liver/kidney function, or hematology were observed after dosing QU (up to 1000 mg/day) for several months in human studies, indicating the considerable tolerance of highly purified QU in the human body and its potential for clinical application [14]. More importantly, growing numbers of recent studies have reported that QU can interact with P-gp and effectively suppress its function, which is expected to be a natural chemosensitizer to enhance the cytotoxic effects of chemotherapeutic drugs on MDR cells [15,16,17,18]. Consequently, as a candidate P-gp inhibitor, QU is worthy of further exploration for its potential to reinforce the potency of antitumor drugs and prevent the occurrence of MDR.

Paclitaxel (PTX) is a tricyclic diterpenoid compound extracted from the bark and needles of Pacific Yew (*Taxus brevifolia*) [19]. As a commonly used first-line chemotherapeutic agent, PTX has successfully treated various carcinomas, such as prostate, lung, ovarian, and breast cancers, by hindering microtubule disassembly and normal mitosis of tumor cells [20,21,22]. Although PTX has improved the overall survival and living quality of cancer patients, the 5-year survival of stage IV breast cancer patients is still very low (circa 20%), which mainly results from the PTX-involved MDR [23]. Thus far, substantial evidence has demonstrated that QU and PTX can synergistically surmount the MDR of drug-resistant tumors and retard cancer cell growth more efficiently than PTX alone as it benefits from the inhibition of P-gp activity by QU [24]. Nevertheless, the codelivery of the above two compounds to tumors in free molecule form still suffers from excessive impediments due to their poor water solubility (PTX: 6 μg/mL, QU: 2.15 μg/mL) and inferior membrane permeability. Therefore, the practical employment of the two therapeutic agents in drug-resistant cancer treatments is severely restricted [25,26,27,28]. With regard to this, it is imperative to introduce strategies for efficiently transporting PTX and QU to tumor regions to fully exert their combined antitumor effects.

**Scheme 1:**
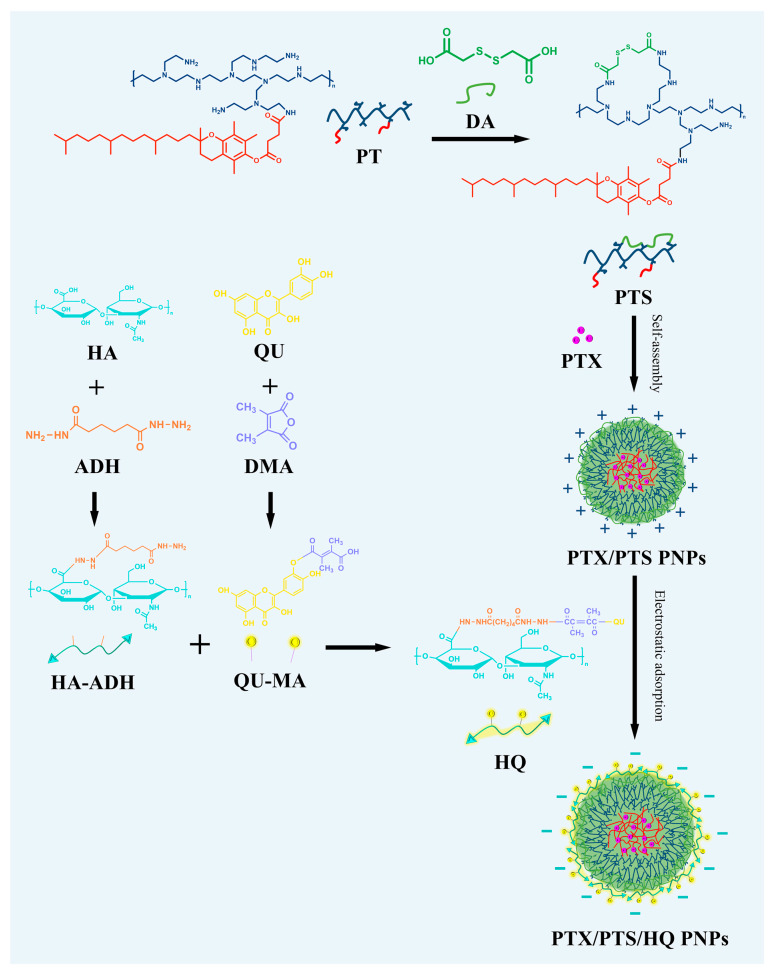
Schematic diagram of the formation process of PTS copolymers, HQ conjugates, PTX/PTS PNPs, and PTX/PTS/HQ PNPs. (Abbreviations: PT, PEI-TOS, polyethyleneimine-tocopherol hydrogen succinate; DA, dithioglycollic acid; PTS, PEI-TOS-SS, polyethyleneimine-tocopherol hydrogen succinate-dithioglycollic acid; PTX, paclitaxel; HA, hyaluronic acid; QU, quercetin; ADH, adipic acid dihydrazide; DMA, 2,3-dimethylmaleic anhydride; HA-ADH, hyaluronic acid-adipic acid dihydrazide; QU-MA, DMA-modified quercetin; HQ, HA-QU, hyaluronic acid-quercetin; PTX/PTS PNPs, PTX/PEI-TOS-SS PNPs, paclitaxel/polyethyleneimine-tocopherol hydrogen succinate-dithioglycollic acid polymeric nanoparticles; PTX/PTS/HQ PNPs, PTX/PEI-TOS-SS/HA-QU PNPs, paclitaxel/polyethyleneimine-tocopherol hydrogen succinate-dithioglycollic acid/hyaluronic acid-quercetin polymeric nanoparticles.)

Polymeric nanoparticles (PNPs), i.e., submicron-sized (10–300 nm) colloidal particles formed in selective solvents, play a key role in the simultaneous conveyance of diverse therapeutic agents to cancer cells [29]. In general, some insoluble chemotherapeutics can be encapsulated into the inner lipophilic portion of PNPs via hydrophobic interactions, while other active compounds can be adsorbed, bound, or conjugated to the surface-functionalized hydrophilic part [30]. Moreover, inspired by some specific hallmarks in the tumor cells, such as reduced pH value (6.2–6.9), overproduced glutathione (GSH, 2–20 mM), and elevated temperature (40–42 °C) [31], multifarious stimuli-responsive PNPs have been developed for enhanced anticancer efficiency by controlling drug release rapidly at tumor sites [32]. Keeping all this in mind, we designed and prepared intracellular stimuli-triggered functionalized hybrid PNPs for co-loading PTX and QU (Scheme 1) which consist of PTX/polyethyleneimine-tocopherol hydrogen succinate-dithioglycollic acid PNPs (PTX/PEI-TOS-SS PNPs, PTX/PTS PNPs) and hyaluronic acid-QU (HA-QU, HQ) conjugates. Since the disulfide bonds (-SS-) of dithioglycollic acid (DA) are sensitive to GSH and the β-carboxylic amide bonds of HQ conjugates are sensitive to pH, we hypothesized that the resultant PTX/PTS/HQ PNPs are capable of achieving specific tumor-targeting and releasing two drugs under an intracellular acidic/redox environment to fully exert synergic antitumor effects, as well as utilizing the cooperation between anticarcinogens and carrier materials to amplify the chemotherapeutic therapy effects in drug-resistant breast cancer. Overall, this work is expected to provide new insight into the coadministration of natural compounds and chemotherapeutic agents for satisfactory combination therapy in MDR cancer.

## 2. Materials and Methods

### 2.1. Materials

Polyethyleneimine (PEI, 25 kDa), tocopherol hydrogen succinate (TOS), DA, deuterium oxide (D_2_O), deuterated methanol (CD_3_OD), 1-ethyl-3 (3-dimethylaminopropyl) carbodiimide (EDC), N-hydroxy succinimide (NHS), and GSH were purchased from Aladdin Reagent Inc. (Shanghai, China). HA (11 kDa) was obtained from Bloomage Freda Biopharm Co., Ltd. (Shandong, China). PTX and QU were provided by Natural Field Biotechnique Co., Ltd. (Shaanxi, China). Dimethyl sulfoxide (DMSO), ethyl acetate, and petroleum benzine were obtained from Sinopharm Chemical Reagent Co., Ltd. (Shanghai, China). All other chemicals were of analytical grade. Dulbecco’s modified Eagle’s medium (DMEM) and LysoTracker Green were purchased from Thermo-Fisher Biochemical Product (Waltham, MA, USA). Rabbit anti-P-gp and mouse anti-β-actin antibodies were purchased from Abcam (Shanghai, China). All other biological reagents were used as received. Human breast cancer cell lines MDA-MB-231, MCF-7, and doxorubicin (DOX)-resistant MCF-7/ADR were purchased from the Institute of Biochemistry and Cell Biology, SIBS, CAS (Shanghai, China). Another DOX-resistant cell lines MDA-MB-231/DOX were induced by our research group through the low-concentration dosage continuous induction method.

### 2.2. Synthesis of Polyethyleneimine-Tocopherol Hydrogen Succinate-Dithioglycollic Acid (PEI-TOS-SS, PTS) Copolymers

PTS copolymers were synthesized through a pre-crosslinking method using DA as linkers between amino groups of the PEI chain. Briefly, DA (18 mg) was activated by EDC (29 mg) and NHS (18 mg) in water at 25 °C for 15 min followed by the addition of a polyethyleneimine-tocopherol hydrogen succinate (PEI-TOS, PT) water solution (2 mg/mL) with gentle stirring for 24 h in darkness. The reaction mixture was then dialyzed to remove unbound materials and small molecules using a dialysis bag (3500 Da) against pure water for 48 h, and the final products were obtained by lyophilization (−80 °C, 1 Pa).

### 2.3. Preparation and Characterization of PTX/PTS/HQ PNPs

PTX/PTS/HQ PNPs were prepared through the sonication method. To obtain PTX/PTS PNPs, PTS copolymers (DS% = 19.8%, 10 mg) were dissolved in pure water (10 mL) and stirred thoroughly. Then, a PTX ethanol solution (18 mg/mL) was added dropwise into the PTS solution with stirring for 30 min, followed by sonication (45 min, 300 W) using a probe-type sonicator (JY92-2D, Ningbo Scientz Biotechnology Co., Ltd., Ningbo, China) in an ice bath. The mixture was then dialyzed toward pure water for 12 h and centrifuged at 3500 rpm for 10 min to remove ethanol and unloaded drugs. Finally, PTX/PTS/HQ PNPs were obtained after the addition of an HQ solution (1 mg/mL) with stirring for 5 min. PTX/PTS/HA PNPs were also prepared using the protocol above but HA was used to surround the cationic PTS instead of HQ. Finally, transmission electron microscopy (TEM, JEM-2010F, Tokyo, Japan) was applied to determine the morphology with an accelerating voltage of 200 kV after staining with 2% phosphotungstic acid. The particle size distribution, zeta potential, and polydispersity index (PDI) were also measured by dynamic light scattering (DLS, Malvern instruments, Malvern, UK).

### 2.4. In Vitro pH/GSH Dual-Stimuli Responsive Drug Release

The pH/GSH-triggered release of QU and PTX from PTX/PTS/HQ PNPs was investigated by a combination of dialysis and the sediment method. Briefly, three different release media: pH 7.4 PBS, pH 6.5 PBS, and pH 5.0 PBS (with 40 mM GSH) were used to mimic the normal physiological milieu and the tumor extracellular/intracellular microenvironment. Then, 9 mL of PTX/PTS/HQ PNPs solutions were poured into a dialysis bag (3500 Da) immersed in 360 mL dissolution media, to achieve sink conditions, and then incubated at 37 °C with mild shaking (120 rpm). For the QU-release study, 1% Tween 80 was employed in media solutions. At each predetermined time point (2, 4, 6, 8, 10, 12, 14, 16, 24, and 36 h), 1 mL of the release medium was withdrawn and replaced with an equal volume of fresh release medium. Then the amount of QU in each collected sample was determined by a high-performance liquid chromatography (HPLC) assay as described in Supplementary Materials 1.8. For the PTX release study, samples collected at each time point were centrifuged (4000 rpm, 10 min) and the precipitates were further dissolved using 1 mL acetonitrile. The solution was next diluted with the mobile phase and the amount of PTX was determined by HPLC analysis as described in Supplementary Materials 1.8. Finally, the cumulative amount of released drugs was calculated, and the percentages of drugs released from PNPs were plotted against time. Similar operations were applied to the GSH- or pH-triggered PTX/QU release behaviors of PTX/PTS PNPs and HQ conjugates.

### 2.5. In Vitro Cellular Uptake (Fluorescence Study)

To qualitatively investigate the cellular internalization of PTX/PTS/HQ PNPs, coumarin-6 (C_6_, Ex = 466 nm, Em = 504 nm) was used as a lipophilic fluorescent probe to substitute PTX for the preparation of drug-loaded PNPs. Specifically, 1 × 10^5^ cells/well of MDA-MB-231/DOX cells were cultured in a glass bottom dish and kept in the incubator overnight for attachment. Then the culture medium was replaced with DMEM containing free C_6_, C_6_/PTS/HA PNPs, and C_6_/PTS/HQ PNPs at equivalent concentrations of 0.1 µg/mL C_6_ at pH 7.4 and 6.5, respectively. After incubation for 4 h, the cells were rinsed with PBS and fixed with cold 4% paraformaldehyde solution for 15 min; subsequently, 6-diamidino-2-phenylindole (DAPI, Ex = 360 nm, Em = 454 nm) was added in the dark to counterstain the nuclei. The cells were then observed using confocal laser scanning microscopy (CLSM, Leica, Wetzler, Germany).

### 2.6. In Vitro Cellular Uptake (HPLC Analysis)

To quantitatively investigate the cellular internalization of PTX/PTS/HQ PNPs, intracellular PTX contents were detected through HPLC in a pH 6.5 milieu. First, MCF-7/ADR cells were seeded in six-well plates (1 × 10^5^ cells/well) and incubated overnight. The cells were then treated with PTX suspension, PTX/PTS/HA PNPs, and PTX/PTS/HQ PNPs (equal to 20 µg/mL PTX). At the designated time points (2 h and 4 h after incubation), the cells were washed thoroughly with PBS, followed by trypsinization and centrifugation (1000 rpm, 3 min). After, the cells were frozen and thawed (−80 °C) three times and ultrasonicated for drug extraction. The supernatant was collected, and the concentration of free PTX was measured through HPLC. Meanwhile, the PTX content was normalized to the protein concentrations according to the following equation:

PTX uptake (μg/mg protein) = the intracellular PTX content/the protein content (1)

### 2.7. Deep Penetrating Ability in Three-Dimensional (3D) Tumor Spheroids

When the size of MCF-7/ADR tumor spheroids reached approximately 600 μm in diameter, free C_6_, C_6_/PTS/HA PNPs and C_6_/PTS/HQ PNPs (equal to 20 µg/mL C_6_) were added and incubated for 12 h. Then, the spheroids were washed thrice with PBS to discard unbound objects and placed in glass bottom dishes. The fluorescent signal of C_6_ in spheroids was observed using the Z-stacking mode of CLSM and surface-plot images were obtained via ImageJ software.

### 2.8. In Vivo Biodistribution Imaging

The subcutaneous MCF-7/ADR human breast tumor-bearing nude mouse model was obtained as described in Supplementary Materials 1.2. Real-time optical imaging experiments were employed to monitor the biodistribution of drug-loaded PTS/HQ PNPs. 1,1′-dioctadecyl-3,3,3′, 3′-tetramethylindotricarbocyanine iodides (DiR, Ex = 748 nm, Em = 780 nm), a near-infrared fluorescence dye, was encapsulated into PTS/HA and PTS/HQ blank PNPs to replace PTX. When the volume of tumors reached approximately 200 mm^3^, mice were randomly divided into three groups (three mice per group) and intravenously injected with free DiR, DiR/PTS/HA PNPs, or DiR/PTS/HQ PNPs (equal to 150 µg/kg DiR). At predetermined time points (1, 4, 7, and 24 h), fluorescence images were taken using an IVIS Lumina Series III (PerkinElmer, Waltham, MA, USA). At 24 h post-injection, mice were euthanized to obtain tumors and major organs, including the heart, liver, spleen, lungs, and kidneys, for ex vivo imaging using the same system. Instrument software was applied to analyze the mean fluorescence intensity of each organ and tumor.

### 2.9. In Vivo Antitumor Efficacy

When MCF-7/ADR tumors grew to approximately 100 mm^3^, the mice were randomly divided into four experimental groups (seven mice per group) and intravenously administered 0.9% saline (as a control), Taxol, PTX/PTS/HA PNPs, or PTX/PTS/HQ PNPs (equal to 5 mg/kg PTX) at 2-day intervals five times. Taxol was prepared according to our previously reported method [33]. Tumor volumes and body weights of mice were monitored every 3 days. At the end of treatments, mice were euthanized to obtain tumors and major organs for subsequent histological analysis.

### 2.10. Statistical Analysis

All experiments were repeated at least three times, and the data are presented as the mean values ± SD. Statistical analyses were performed using the student’s t-test and one-way analysis of variance (ANOVA). A *p*-value < 0.05 was considered statistically significant.

## 3. Results and Discussion

### 3.1. Synergetic Cytotoxicity Effects of PTX and QU

**Figure 1:**
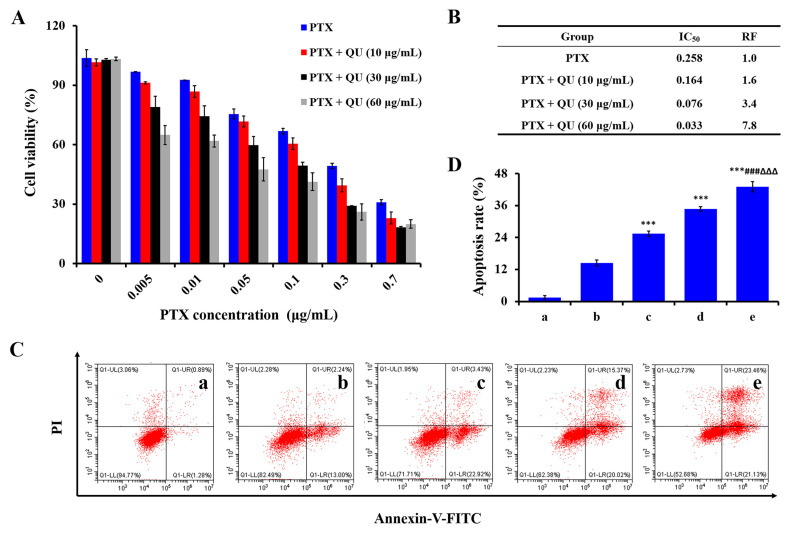
The synergetic cytotoxic effect of PTX and QU. (**A**) The viability of MCF-7/ADR breast cancer cells after treatment with PTX alone or in combination with increasing concentrations of QU for 72 h. (**B**) The summarized table of IC_50_ (μg/mL) and RF values. (**C**) Flow cytometric analysis of cell apoptosis after treatment with (**a**) DMEM, (**b**–**e**) free PTX at 0.3 μg/mL in combination with QU at different concentrations (0, 10, 30, and 60 μg/mL) for 72 h. (**D**) Quantitative analysis of the total apoptosis rate. Statistical significance: *** *p* < 0.005 compared with the b group, ^###^ *p* < 0.005 compared with the c group, ^∆∆∆^ *p* < 0.005 compared with the d group. Data are presented as the mean values ± SD (*n* = 3).

The in vitro cytotoxicity of free PTX combined with QU in MCF-7/ADR cells was determined by the MTS method. As shown in Figure 1A, all groups inhibited the proliferation of cells in a dose-dependent manner. The half-maximal inhibitory concentrations (IC_50_) of PTX with various concentrations of QU (0, 10, 30, and 60 μg/mL) listed in Figure 1B were 0.258 μg/mL, 0.164 μg/mL, 0.076 μg/mL, and 0.033 μg/mL, respectively. Correspondingly, the reversal fold (RF) values of the three combination groups were 1.6, 3.4, and 7.8, respectively; indicating that QU can elevate the toxicity of PTX to MCF-7/ADR cells. According to Figure 1C,D, the collaboration of free PTX and QU (10, 30, and 60 μg/mL) led to a promoted total cell apoptosis rate (25.5%, 34.8%, and 43.1%) compared with PTX alone (14.5%), further confirming the synergetic anticancer efficacy of the two active components.

### 3.2. Preparation, Characterization, and Responsive Behaviors of PTS Copolymers and HQ Conjugates

#### 3.2.1. Preparation and Characterization of PTS Copolymers and HQ Conjugates

**Figure 2:**
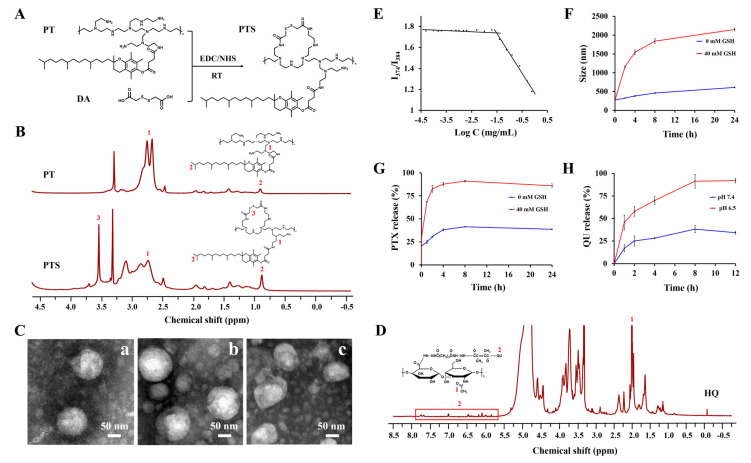
The preparation, characterization, and physiochemical properties of PTS copolymers and HQ conjugates. (**A**) Synthesis route of PTS copolymers. (**B**) ^1^H NMR spectra of PT and PTS copolymers. (**C**) TEM images of (**a**) PT, (**b**) PTS, and (**c**) PTS/HA blank PNPs. (**D**) ^1^H NMR spectra of HQ conjugates. (**E**) Intensity ratio plot of I_374_/I_384_ of pyrene versus log C for PTS copolymers. The change in (**F**) size and (**G**) PTX release of PTX/PTS PNPs treated with or without GSH. (**H**) QU release from HQ conjugates at pH 6.5 and pH 7.4. Data are presented as the mean values ± SD (*n* = 3).

As depicted in Section 2.2. and Figure 2A, the -SS- of DA were introduced into the preparation process of PT copolymers to obtain the resultant PTS copolymers. In the ^1^H-nuclear magnetic resonance (^1^H NMR) spectra of PT and PTS copolymers (Figure 2B), PEI and TOS were characterized by the peaks of -CH_2_- and -CH(CH_3_)_2_ protons at (1) 2.5–3.0 ppm and (2) 0.8 ppm, respectively. In addition, new peaks arising at (3) 3.5–3.7 ppm corresponding to the -CH_2_- protons of DA were observed, indicating the successful synthesis of PTS copolymers. The formation of HQ conjugates was also confirmed by ^1^HNMR (Figure 2D). The peaks located at (1) 1.9–2.1 ppm were ascribed to the chemical shifts of -CH_3_ in HA, and the proton signals of QU (the chemical structure is shown in Figure S3) were assigned as follows (2): 5.8–6.2 ppm (6H, 8H), 7.0 ppm (2′H), and 7.5–7.8 ppm (5′H, 6′H).

#### 3.2.2. Critical Micelle Concentration (CMC) Determination of PTS Copolymers

CMC is an important parameter to describe the self-assembly behavior of amphiphilic polymers and the dilution stability of a system [34]. As presented in Figure 2E, the CMC value of PTS copolymers was 30.72 μg/mL, indicating that the copolymers can self-assemble into micelles at low concentrations in aqueous solutions. Moreover, such a low CMC value also implied that PTS PNPs had a good antidilution ability which is beneficial for maintaining their structural stability to avoid drug leakage during blood circulation.

#### 3.2.3. Preparation and Characterization of PT, PTS, and PTS/HA Blank PNPs

PT, PTS, and PTS/HA blank PNPs were further prepared through the sonication method. Then, TEM and DLS measurements were utilized to characterize the morphology, size distribution, and zeta potential of each sample. Figure 2C showed that these blank PNPs were spherical in shape and dispersed well with an average diameter of approximately 120–150 nm, which was consistent with the DLS results summarized in Table 1. Generally, the particle size detected by TEM was smaller than that detected by DLS analysis, which was mainly due to the shrinkage of samples during the dehydration process prior to TEM imaging, while the DLS assay measured the hydrodynamic radius of wet samples [35]. Additionally, three blank PNPs exhibited a suitable PDI (0.1–0.2) and modest zeta potential (circa ± 30 mV), implying their good dispersion and stability in an aqueous medium. Meanwhile, the surface charge conversion from electropositivity (PTS blank PNPs) to electronegativity (PTS/HA blank PNPs) revealed the successful introduction of anionic HA.

#### 3.2.4. Selective pH or Redox Sensitivity of HQ Conjugates or PTS Copolymers

We next examined the reductive sensitivity of PTX/PTS PNPs in response to 40 mM GSH (which corresponds to the concentration in the cytoplasm of tumor cells [36]) by monitoring the variations in size and drug release. The experimental results (Figure 2F) showed that the particle size of PTX/PTS PNPs swelled rapidly from ~275 nm to ~1841 nm within 8 h after incubation with 40 mM GSH, while a small size change (from ~275 nm to ~463 nm) was observed in the absence of GSH under the same conditions. Similar characteristics were discovered in the test of PTX release behaviors. As displayed in Figure 2G, PTX/PTS PNPs exhibited fast drug release in a simulative redox environment, and the accumulation of PTX release reached ~90.9% within 8 h which was 2.2-fold than that of the group treated with 0 mM GSH (~41.4%). These phenomena can be interpreted as the breakage of -SS-, which led to a looser structure of PTS PNPs and accelerated the release of PTX. Furthermore, the QU-release ability of HQ conjugates under acidic conditions is illustrated in Figure 2H. In pH 7.4 PBS, approximately 36.3% QU was released within 12 h, while in contrast, a promoted release (~93.7%) was monitored in pH 6.5 PBS medium, demonstrating the pH-responsive property of HQ conjugates.

### 3.3. Characterization and Pharmaceutical Evaluation of PTX/PTS/HQ PNPs

#### 3.3.1. Characterization

**Figure 3:**
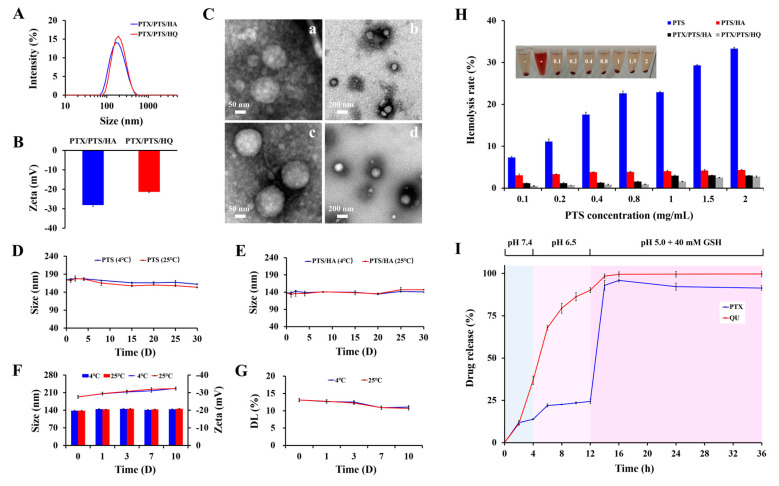
Characterization and pharmaceutical evaluation of PTX/PTS/HQ PNPs. (**A**) Hydrodynamic size distribution and (**B**) zeta potential of PTX/PTS/HA PNPs and PTX/PTS/HQ PNPs. (**C**) Typical TEM images of (**a,b**) PTX/PTS/HA PNPs and (**c,d**) PTX/PTS/HQ PNPs at different bars. Size changes of (**D**) PTS blank PNPs and (**E**) PTS/HA blank PNPs at 4 °C and 25 °C for 30 days. Storage stability of PTX/PTS/HQ PNPs, including (**F**) particle size (line chart), zeta potential (column chart), and (**G**) DL (%) of PTX at 4 °C and 25 °C for 10 days. (**H**) Hemolysis assay of PTS blank PNPs, PTS/HA blank PNPs, PTX/PTS/HA PNPs, and PTX/PTS/HQ PNPs with PTS concentrations in the range of 0.1–2 mg/mL. The inset image represents the hemolysis situation of PTX/PTS/HQ PNPs. (**I**) Dual pH/redox-responsive release profile of QU and PTX from PTX/PTS/HQ PNPs with or without 40 mM GSH at pH 7.4, pH 6.5, and pH 5.0 for 36 h, respectively. Data are presented as the mean values ± SD (*n* = 3).

PTX/PTS/HA PNPs and PTX/PTS/HQ PNPs were fabricated as illustrated in Section 2.3. Meanwhile, physical properties (Figure 3A–C, Table 1), including particle size, PDI, zeta potential, morphology, drug loading (DL), and encapsulation efficiency (EE), were measured via DLS, TEM, and HPLC analysis. First, both formulations had a single and narrow distribution peak with suitable particle diameters (<200 nm) and PDI values (<0.2) which allow these PNPs to penetrate and accumulate in tumor areas through the enhanced permeability and retention (EPR) effect, thus making them conducive for drug delivery [37]. Furthermore, both formulations had negative surface charges (circa −28 mV and −21 mV) due to the existence of anionic HA, which can minimize nonspecific binding with serum proteins and increase hemocompatibility compared to cationic surfaces [38]. In addition, TEM images showed that both formulations possessed a smooth, spherical shape with an average diameter of around 160 nm which matched the DLS data. Finally, the HPLC assay indicated that the DL and EE of PTX in PTX/PTS/HA PNPs were approximately 10.67% and 61.83%, respectively. Moreover, the DL for PTX and QU was calculated to be approximately 9.69% (the EE of PTX was nearly 49.84%) and 1.45% in PTX/PTS/HQ PNPs, respectively.

#### 3.3.2. In Vitro Physical Stability

Stability is one of the most vital factors for nanodrug delivery systems and will provide great guidance for animal administration and clinical application [39]. According to Figure 3D and E, apparent variations were seldom observed in the hydrodynamic size of PTS blank PNPs (~15 nm fluctuation) and PTS/HA blank PNPs (~10 nm fluctuation), suggesting that they can be stored at 4 °C or 25 °C for at least 1 month. Meanwhile, no significant change (Figure 3F,G) was found in particle size (+15 nm or so), zeta potential (+1 mV or so), or PTX loading (−0.5% or so) of PTX/PTS/HQ PNPs at 4 °C and 25 °C during the 3 days of the assay, demonstrating the considerable physical stability of the drug-loaded PNPs.

#### 3.3.3. Hemolysis

Hemocompatibility is an essential property for evaluating the clinical treatment safety of intravenously administered PNPs [40]. According to the stipulation of the American Society for Testing and Materials (ASTM F756-17, 2017), a hemolysis rate of less than 5% is regarded as nontoxic and safe [41]. As demonstrated in Figure 3H, the hemolysis rate of PTS blank PNPs was up to 7.3% at a PTS concentration of only 0.1 mg/mL, indicating the underlying risk for systemic administration. In distinct contrast, a negligible hemolytic effect can be observed after treatment with PTS/HA blank PNPs (2.8–4.3% homolysis), PTX/PTS/HA PNPs (1.1–3.1% homolysis), and PTX/PTS/HQ PNPs (0.5–2.7% homolysis) within the PTS concentration range of 0.1–2 mg/mL, proving the considerable blood compatibility of these PNPs, which are thus feasible for intravenous injection. Additionally, the representative image of the homolysis situation also exhibited an obvious distinction between the positive control group (almost complete hemolysis) and the PTX/PTS/HQ PNPs group (few disruptions to the integrity of the erythrocyte membrane which was similar to the negative control). These results suggest that the modification of HA on the surface of PTS PNPs can improve the hemocompatibility mostly on account of the following two reasons: (1) the reduced destruction effect against the electron-negative cell membrane caused by the cationic polymer PEI [42]; (2) the promoted resistance to oxidative hemolysis, which was due to the antioxidant activity of HA [43].

#### 3.3.4. pH/GSH-Triggered QU and PTX Dual-Drug Release Behaviors

To investigate the potential stimuli-responsive ability of dual-sensitive PTX/PTS/HQ PNPs at pathological sites, in vitro release profiles of QU and PTX were tested in the mimetic microenvironments of normal and tumor tissues. As shown in Figure 3I, the QU (~11.2%)/PTX (~11.9%)-release rate was less than 40% within the experimental time of the initial 2 h at pH 7.4, suggesting that there was no obvious drug release burst of PTX/PTS/HQ PNPs in a normal physiological environment. When the release time was extended to 4 h, the cumulative release rates of QU and PTX reached nearly 36.9% and 13.8%, respectively, implying that the PNPs structures were almost stable during blood circulation with less premature drug release. As the pH decreased from 7.4 to 6.5 over the next 8 h incubation period, the QU release from PTX/PTS/HQ PNPs was remarkably accelerated, with an accumulative release rate reaching up to ~90.1% at 12 h. This phenomenon was consistent with the results of the QU release study described in Figure 2H, which might be ascribed to the pH-triggered cleavage of β-carboxylic amide bonds between HA and QU. The pH sensitivity of β-carboxylic amide bonds can be attributed to the internal attack of the amide carbonyl group by β-carboxylate [44]. In contrast, the PTX release rate (13.8–24.4%) was practically unaffected when PTX/PTS/HQ PNPs were exposed to an acidic environment for the same time period (4–12 h). Under the condition of pH 5.0 with 40 mM GSH, the QU release was again increased slightly due to the continuously reduced pH values of the media, and the cumulative release rate reached approximately 98.4% at 14 h. However, an obvious burst release of PTX was recorded between 12 h (24.4%) and 14 h (~92.9%) in the presence of GSH, suggesting the redox-responsive property of PTX/PTS/HQ PNPs. A similar phenomenon was observed in Figure 2G which was likely attributed to GSH breaking the -SS- into two sulfhydryl groups (–SH) and consequently causing the collapse of the self-assembled PNPs [45]. Overall, these results revealed that PTX/PTS/HQ PNPs performed in an on-demand drug release manner with dual-stimuli responsivity, which would help efficiently deliver anticancer drugs to tumor sites and achieve rapid oncology therapy.

### 3.4. Cellular Internalization and In Vitro Anticancer Efficacy of PTX/PTS/HQ PNPs

#### 3.4.1. Cytocompatibility of Blank PNPs

**Figure 4:**
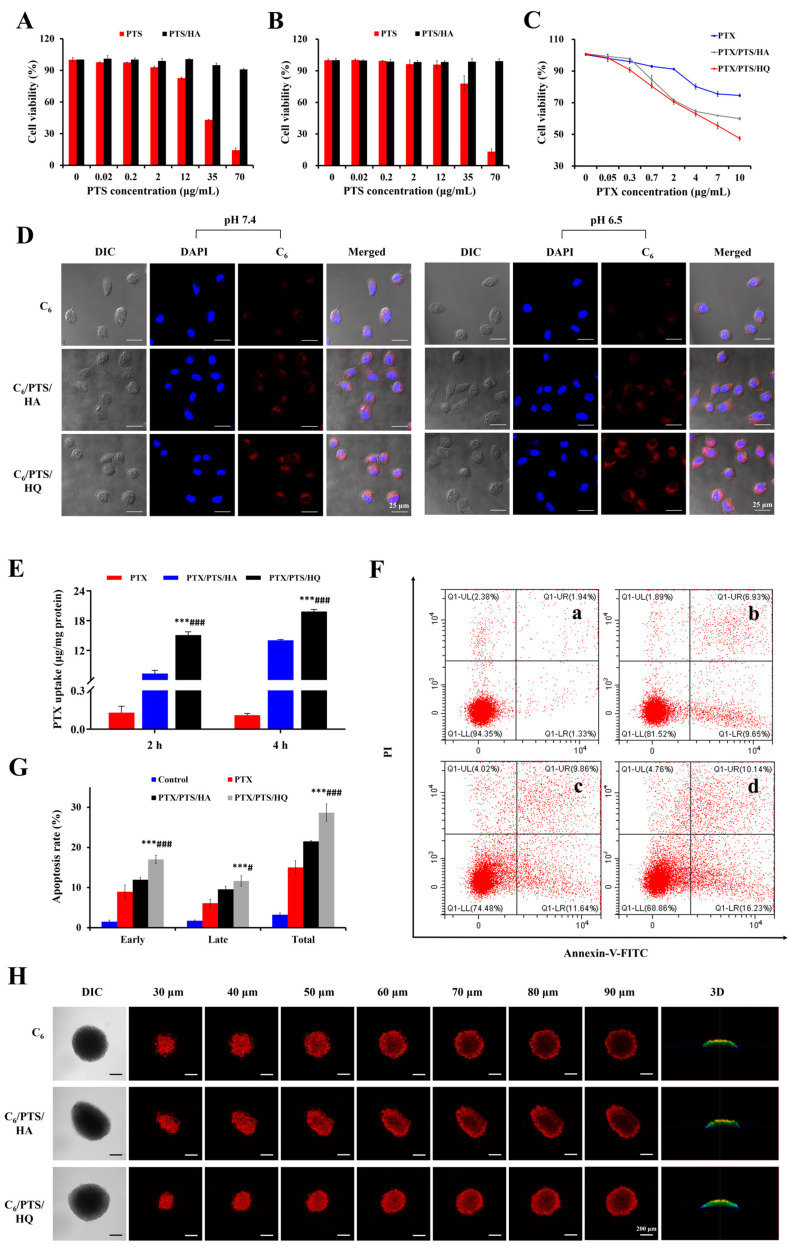
Cellular accumulation and in vitro antitumor therapeutic potential of PTX/PTS/HQ PNPs. Cell viabilities of (**A**) MDA-MB-231/DOX and (**B**) MCF-7/ADR cells incubated with PTS and PTS/HA blank PNPs for 48 h. (**C**) In vitro cytotoxicity profiles of MCF-7/ADR cells cultured with PTX suspension, PTX/PTS/HA PNPs, and PTX/PTS/HQ PNPs for 48 h. (**D**) CLSM images of cellular uptake in MDA-MB-231/DOX cells after treatment with C_6_ suspension, C_6_/PTS/HA PNPs, and C_6_/PTS/HQ PNPs for 6 h at pH 7.4 and pH 6.5. (**E**) Intracellular PTX concentration in MCF-7/ADR cells after treatment with PTX suspension, PTX/PTS/HA PNPs, and PTX/PTS/HQ PNPs at pH 6.5 for 2 h or 4 h. (**F**) Annexin V-FITC apoptosis assay to determine apoptotic/necrotic cells in MCF-7/ADR cells treated with (**a**) DMEM, (**b**) PTX suspension, (**c**) PTX/PTS/HA PNPs, and (**d**) PTX/PTS/HQ PNPs for 48 h by flow cytometry. (**G**) Quantitative analysis of the early, late, and total apoptosis rates. (**H**) Z-stack images of C_6_ suspension, C_6_/PTS/HA PNPs, and C_6_/PTS/HQ PNPs penetrating into the 3D MCF-7/ADR tumor spheroids for 12 h. The surface of the spheroids was defined as 0 µm. Statistical significance: *** *p* < 0.005 compared with the PTX suspension group, ^#^ *p* < 0.05, ^###^ *p* < 0.005 compared with the PTX/PTS/HA PNPs group. Data are presented as the mean values ± SD (*n* = 3).

Excellent cell biocompatibility is an indispensable factor for the potential application of polymeric nanocarriers in biomedical areas [46]. As exhibited in Figure 4A,B, dose-dependent cytotoxicity was observed in two tumor cells after treatment with PTS blank PNPs and the viability of MDA-MB-231/DOX (14.5%) and MCF-7/ADR (13.2%) cell lines was reduced sharply at a PTS concentration of 70 μg/mL. Such toxic effects might be attributed to the high surface positive charges of PEI damaging the integrity of cell membranes [47]. On account of the HA modification on the surface of PTS blank PNPs, the viability of both cell lines (MDA-MB-231/DOX: 90.9%; MCF-7/ADR: 99.2%) was above 90%, even at a high PTS concentration of 70 μg/mL, indicating the favorable safety of PTS/HA blank PNPs for the intracellular delivery of anticancer agents.

#### 3.4.2. Cytotoxicity Test

To demonstrate the inhibitory effect of PTX/PTS/HQ PNPs on MDR breast cancer cell proliferation, cytotoxicity experiments were conducted against MCF-7/ADR cell lines through the MTS method. As shown in Figure 4C, the cytotoxic effect of the three groups was distinctly enhanced as the PTX concentration increased from 0.05 μg/mL to 10 μg/mL after 48-h incubation. Specifically, PTX/PTS/HQ PNPs showed significant inhibition of cellular proliferation with a reduced IC_50_ value (9.348 μg/mL) compared to PTX suspension (44.377 μg/mL) and PTX/PTS/HA PNPs (16.240 μg/mL) groups. These results illustrated that PTX/PTS/HQ PNPs can elevate the sensitivity of the chemotherapeutic drug PTX to drug-resistant cancer cells and consequently achieve a better anticancer effect in vitro.

#### 3.4.3. Cell Apoptosis Assay

A flow cytometry assay was subsequently applied to investigate the apoptosis-inducing capability of PTX/PTS/HQ PNPs on MCF-7/ADR cells. According to Figure 4F,G, negligible apoptotic cells were detected in the control group at 48 h, with a total apoptosis rate of approximately 3.2%. By comparison, the total cell apoptosis proportions of MCF-7/ADR cells after culture with free PTX, PTX/PTS/HA PNPs, and PTX/PTS/HQ PNPs were 15.0%, 21.5%, and 28.7%, respectively. The highest percentage of early, late, and total apoptotic cells was obtained after treatment with PTX/PTS/HQ PNPs, confirming the enhanced growth inhibition efficacy of the drug codelivery system on MDR tumor cells. The cell apoptosis effects of PTX/PTS/HQ PNPs were consistent with the above cytotoxicity results and were likely one of the reasons for their potent anticancer efficacy on drug-resistant cancer cells.

#### 3.4.4. Intracellular Uptake

The cellular uptake in MDA-MB-231/DOX and MCF-7/ADR cell lines was examined qualitatively and quantitatively using CLSM and HPLC analysis, respectively. As demonstrated in Figure 4D, the nuclei stained with DAPI presented blue fluorescence, while the red fluorescence was from C_6_ (a fluorescent marker) which was evenly distributed in the cytoplasm. Under pH 7.4 conditions, weak red fluorescence could be detected in the free C_6_-treated group after 4 h of incubation, indicating a low intracellular uptake ability. Conversely, stronger fluorescence signals were clearly observed when cells were exposed to C_6_/PTS/HA PNPs or C_6_/PTS/HQ PNPs which could be explained by the specific binding between HA and CD44 receptors (an adhesion protein overexpressed on the surface of various carcinoma cells) [48]. Meanwhile, a similar tendency was monitored under pH 6.5 incubation conditions, but the red fluorescence of C_6_/PTS/HQ PNPs was brighter than that in pH 7.4 medium. Quantitative uptake results (Figure 4E) also showed that the intracellular PTX concentration in the PTX/PTS/HA PNPs and PTX/PTS/HQ PNPs groups was higher (2 h: 56.31- and 116.08-fold, respectively; 4 h: 127.6- and 180.3-fold, respectively) than that in the free PTX group in an acidic environment. Collectively, these results reflected that PTX/PTX/HQ PNPs can respond to a weakly acidic environment and realize satisfactory QU release (consistent with the data described in Figure 3I) to reverse drug efflux effects, thus achieving efficient PTX cellular uptake in MDR breast cancer cells.

#### 3.4.5. Penetration into 3D Tumor Spheroids

Tumor spheroids are considered to be an ideal 3D model to simulate the complex pathology environment in vivo due to their increased cell density, enhanced interstitial pressure, and diverse tumor perfusion [49] which were utilized in this study to assess the penetration capacity of drug-loaded PTS/HQ PNPs deep into tumor tissues. From CLSM graphics (Figure 4H), the red fluorescence detected in C_6_/PTS/HA PNPs and C_6_/PTS/HQ PNPs groups were stronger than that of the free C_6_ group. Especially at a scanning depth of 90 µm, C_6_/PTS/HQ PNPs exhibited the highest fluorescence signals which even appeared in the center of tumor spheroids. These results validated that drug loaded PNPs can efficiently prompt the permeation of “cargo” into pathological sites through the EPR effect which is advantageous for MDR tumor treatments.

### 3.5. Lysosomal Escape, HA Targeting, and P-Gp Inhibition of Drug-Loaded PTS/HQ PNPs

#### 3.5.1. Lysosomal Escape

**Figure 5:**
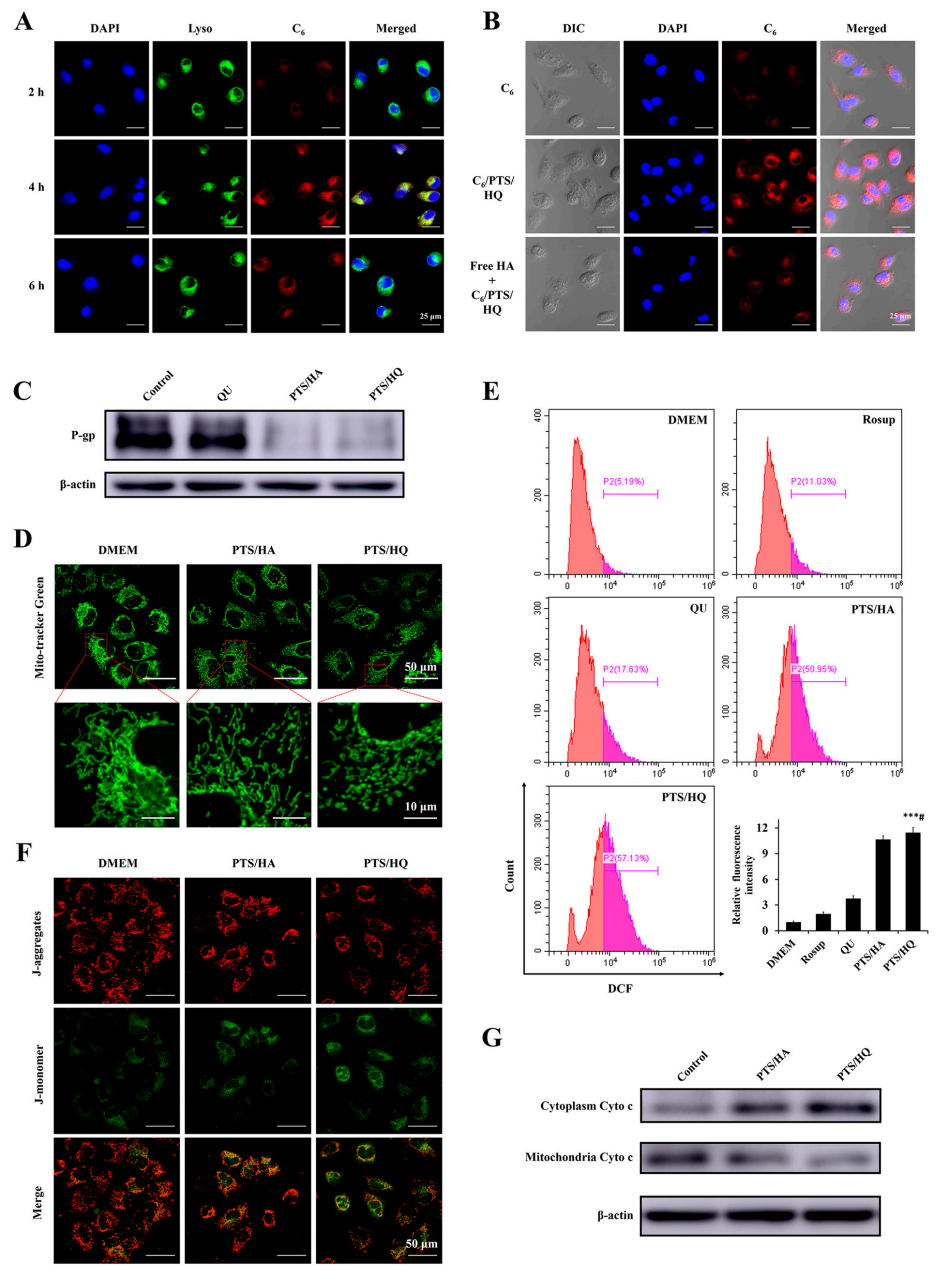
Intracellular mechanisms of drug-loaded PTS/HQ PNPs to combat MDR. (**A**) Lysosomal escape of C_6_/PTS/HQ PNPs observed in MDA-MB-231/DOX cells after 2, 4, and 6 h of treatment. Nuclei and lysosomes were stained with DAPI (blue) and Lyso Tracker (green), respectively. (**B**) Cellular uptake of C_6_ by MDA-MB-231/DOX cells after 4 h incubation with C_6_ suspension, C_6_/PTS/HQ PNPs, and free HA (pretreated for 2 h) + C_6_/PTS/HQ PNPs. (**C**) Western blot analysis of P-gp expression on MDA-MB-231/DOX cells after treatment with free QU, PTS/HA blank PNPs, and PTS/HQ blank PNPs for 24 h. (**D**) Mitochondrial morphology, (**E**) ROS generation, (**F**) mitochondrial depolarization, and (**G**) Cyto c levels in MCF-7/ADR cells after treatment with PTS/HA blank PNPs and PTS/HQ blank PNPs for 24 h. Statistical significance: *** *p* < 0.005 compared with the QU group, ^#^ *p* < 0.05 compared with the PTS/HA blank PNPs group. Data are presented as the mean values ± SD (*n* = 3).

Long-term retention in lysosomes is unbeneficial for PNPs to exert drug efficacy in specific organelles after internalization into tumor cells [50]. An intracellular colocalization experiment was conducted in MDA-MB-231/DOX cell lines to gain insight into the lysosomal escape capability of drug-loaded PTS/HQ PNPs. As indicated by CLSM images (Figure 5A), merged yellow signals (green signals of Lysotracker Green overlapped with red signals of C_6_) were observed after 4 h of treatment, indicating a considerable cellular uptake ability of C_6_/PTS/HQ PNPs. However, the overlapping fluorescence was significantly reduced at the 6 h point, suggesting a gradual escape of C_6_/PTS/HQ PNPs from lysosomal compartments to the cytoplasm which was likely caused by the “proton sponge effects” of PEI [51].

#### 3.5.2. HA-Targeted Cellular Internalization

The HA-competitive inhibition test was performed in MDA-MB-231/DOX cells to qualitatively evaluate the HA-targeted endocytosis capacity of C_6_/PTS/HQ PNPs. As presented in Figure 5B, C_6_ fluorescence signals from the C_6_/PTS/HQ PNPs group were stronger than those of the free C_6_ and HA-pretreated C_6_/PTS/HQ PNPs groups after 4 h of incubation. This phenomenon demonstrated that the enhanced drug-cellular uptake exhibited in Figure 4D was attributed to the specific CD44-HA affinity, which could be competitively restrained by free HA through preoccupying partial CD44 receptors. Hence, we concluded that the active tumor-targeting specificity of drug-loaded PTS/HQ PNPs could boost the intracellular accumulation of therapeutic agents, thus contributing to achieving appreciable therapeutic effects on MDR tumors.

#### 3.5.3. P-Gp Inhibition

One of the drug-resistance mechanisms of MDA-MB-231/DOX cell lines is P-gp overexpression, as confirmed in our preliminary experiments (Figure S4). Herein, the P-gp inhibition effect of drug-loaded PTS/HQ PNPs was evaluated in MDA-MB-231/DOX cells through the western blot analysis. According to Figure 5C, free QU, PTS/HA PNPs, and PTS/HQ PNPs-treated groups all downregulated P-gp protein levels compared with the control group. In particular, PTS/HA and PTS/HQ PNPs exhibited a remarkable decrease in P-gp expression, thus alleviating the chemotherapeutics-efflux effect and augmenting the intracellular accumulation of the loaded drug (Figure 4D,E), which was likely due to both QU and TOS used in our nanoparticle carriers possessing P-gp inhibitory activity [52].

### 3.6. Mitochondrial Function Adjustment of Drug-Loaded PTS/HQ PNPs

Mitochondria play essential roles in the regulation of energy metabolism and apoptotic signaling pathways, emerging as an effective target organelle for MDR cancer therapy [53]. TOS and QU can specifically cause mitochondrial dysfunction by stimulating the production of reactive oxygen species (ROS), activating stress proteins, and so on [54]. Hence, a series of tests were conducted to investigate the effects of drug-loaded PTS/HQ PNPs on mitochondrial biological functions.

#### 3.6.1. Morphological Assay of Mitochondria

From the CLSM image (Figure 5D), the mitochondria of untreated cells are rod-like and form an intact and compact reticular structure. However, the mitochondrial network collapsed after cells were exposed to PTS/HA blank PNPs. Similar phenomena appeared in the PTS/HQ blank PNPs group and the amounts of fragmented mitochondria (dotlike) were increased, indicating the severe damage of PTS/HQ blank PNPs on mitochondrial morphology.

#### 3.6.2. Detection of ROS Levels

Overproduced ROS can not only inhibit the mitochondrial respiratory chain but also impair the mitochondrial membrane [55]. Because nonfluorescent 2′,7′-dichlorodihydrofluorescein diacetate (DCFH-DA) dye can be oxidized by ROS to produce fluorescent 2′,7′-dichlorofluorescein (DCF) [56], intracellular ROS levels were monitored by quantifying DCF fluorescence using flow cytometry. According to Figure 5E, the amount of ROS generated in the PTS/HQ blank PNPs-treated group was 5.8-, 3.0-, and 1.1-fold higher than that in the Rosup (positive control), free QU, and PTS/HA blank PNPs groups, respectively, demonstrating that PTS/HQ blank PNPs indeed promoted the production of endogenous ROS.

#### 3.6.3. Mitochondrial Membrane Potential (MMP) Analysis

In response to elevated ROS levels, the mitochondrial permeability transition pores (MPTPs) will open and cause a decrease in the MMP which can be characterized by the fluorescence switch of 5,5′,6,6′-tetrachloro-1,1′,3,3′-tetraethylbenzimidazolyl carbocyanine iodide (JC-1) from red (JC-1 aggregates) to green (JC-1 monomers) [57]. As revealed in Figure 5F, more green fluorescence was detected in PTS/HQ blank PNPs-treated cells than in untreated and PTS/HA blank PNPs-treated cells, demonstrating the MMP-decreasing effects of PTS/HQ blank PNPs.

#### 3.6.4. Cytochrome c (Cyto c) Release

One of the hallmarks of mitochondrial damage is increased outer membrane permeabilization (OMP), followed by the release of Cyto c from intermembrane spaces (IMS) into the cytosol [58]. From the western blot images (Figure 5G), both PTS/HA and PTS/HQ blank PNPs increased the expression of cytoplasmic Cyto c while concomitantly decreasing the mitochondrial Cyto c level compared to the control group. PTS/HQ blank PNPs exhibited the highest and lowest protein expression of Cyto c in the cytoplasm and mitochondria, respectively, demonstrating its effective mitochondrial destruction ability.

### 3.7. In Vivo Biodistribution Study

**Figure 6:**
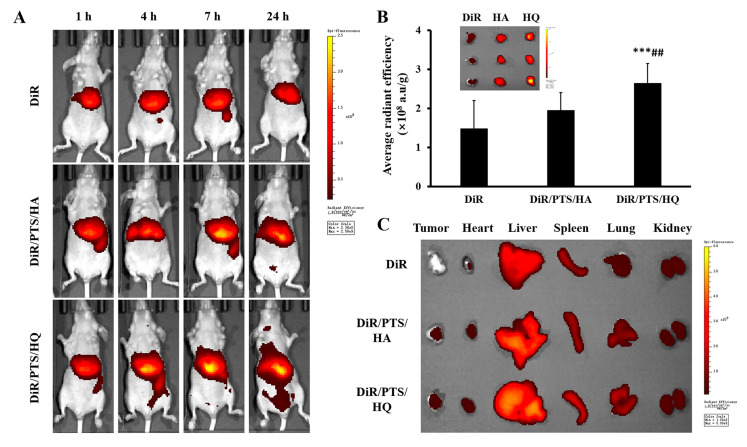
In vivo tumor-targeting evaluation of drug-loaded PTS/HQ PNPs. (**A**) In vivo fluorescence images of DiR suspension, DiR/PTS/HA PNPs, and DiR/PTS/HQ PNPs after tail vein injection into MCF-7/ADR-bearing nude mice for 1, 4, 7, and 24 h. (**B**) Quantitative analysis of the fluorescence intensities of DiR in tumors. Ex vivo images of isolated tumors are also shown in the inset figure. (**C**) Images of isolated tumors and organs at 24 h post-injection. Statistical significance: *** *p* < 0.005 compared with the free DiR group, ^##^ *p* < 0.01 compared with the DiR/PTS/HA PNPs group. Data are presented as the mean values ± SD (*n* = 3).

The fluorescence distribution of DiR was tracked in vivo to evaluate the tumor-targeting ability of drug-loaded PTS/HQ PNPs in MCF-7/ADR tumor-bearing nude mice. According to whole-body images (Figure 6A), the fluorescence intensity at tumor sites gradually increased over time and reached a maximum at 24 h in the DiR/PTS/HA PNPs and DiR/PTS/HQ PNPs groups, whereas the fluorescent signal of tumors was barely detectable in the free DiR group. Quantitative analysis (Figure 6B) of excised tumors at 24 h post-injection further demonstrated that the DiR/PTS/HQ PNPs group displayed the strongest fluorescence accumulation which was 1.79- and 1.36-fold higher than that of the free DiR and DiR/PTS/HA PNPs groups, respectively. These results suggest that drug-loaded PTS/HQ PNPs had both passive and active targeting capacity via the EPR effect and specific binding effect between HA and CD44 receptors, respectively. Ex vivo fluorescence imaging of isolated organs was also examined, as presented in Figure 6C. DiR accumulation in livers and spleens was likely due to rapid clearance by resident macrophages according to published reports [59].

### 3.8. In Vivo Antitumor Activity and Biosafety Evaluation of PTX/PTS/HQ PNPs

#### 3.8.1. In Vivo Antitumor Activity

**Figure 7:**
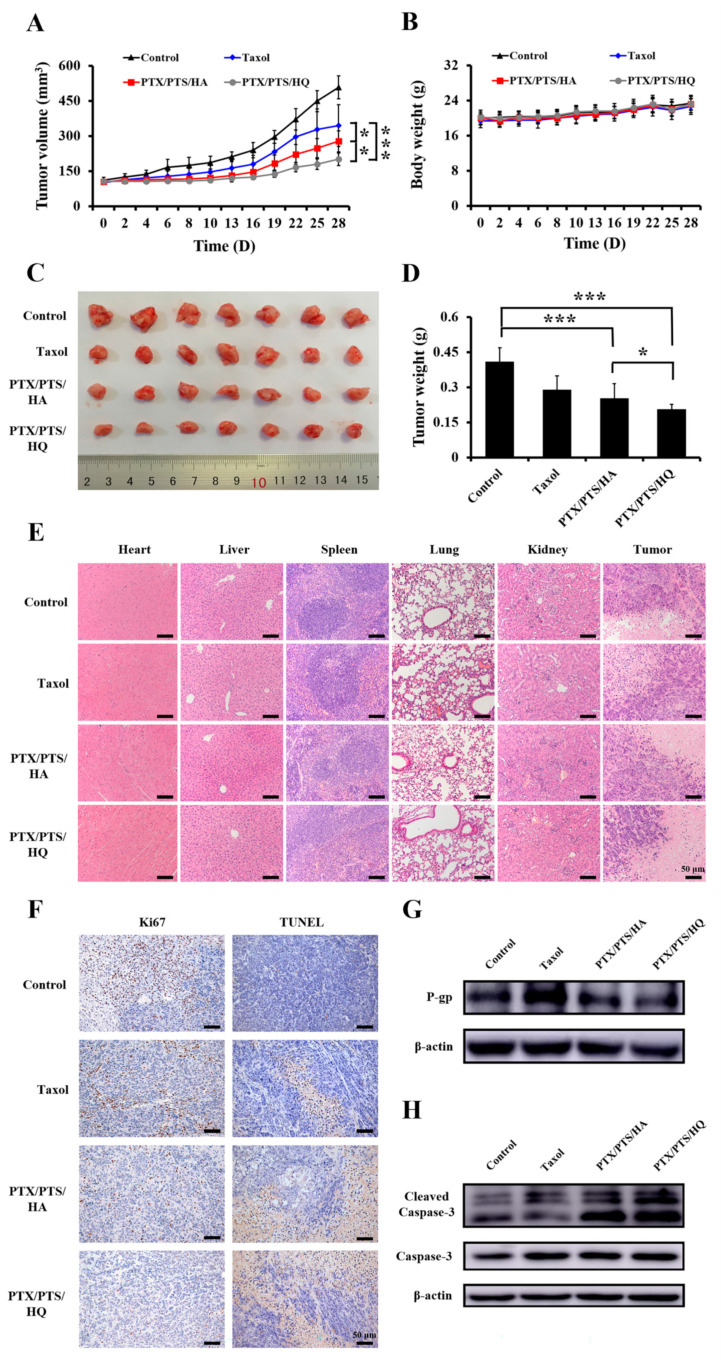
In vivo antitumor therapeutic efficacy of PTX/PTS/HQ PNPs in MCF-7/ADR tumor-bearing nude mice. (**A**) Tumor volume evolution and (**B**) body weight changes within the 28-day observation period. (**C**) Images and (**D**) average weights of dissected solid tumors from the treated mice at the end of experiments. (**E**) H&E staining assay of the tumor and main organs extracted from tumor-bearing nude mice. (**F**) Ki67 immunohistochemistry and TUNEL staining of tumor tissues. Brown areas indicate positive results of proliferative cells and apoptotic cells. (**G**) P-gp, (**H**) Caspase-3 and cleaved Caspase-3 protein expression in excised tumors by western blot assay. Statistical significance: * *p* < 0.05, ** *p* <0.01, *** *p* < 0.005. Data are presented as the mean values ± SD (*n* = 7).

The in vivo anticancer efficiency of PTX/PTS/HQ PNPs was evaluated in a subcutaneous MCF-7/ADR human breast tumor-bearing nude mouse model. As exhibited in Figure 7A, PTX/PTS/HQ PNPs significantly slowed down tumor growth and the average tumor volume in the PTX/PTS/HQ PNPs group was 2.54-, 1.72-, and 1.28-fold smaller than that of control, Taxol, and PTX/PTS/HA PNPs groups at day 28, respectively. In addition, negligible variation in body weights (Figure 7B) was observed in PTX/PTS/HQ PNPs-treated mice during the experimental period, implying the low toxicity and good tolerability of PTX/PTS/HQ PNPs. At the endpoint of experiments, tumors were harvested, imaged, and weighed. The visual observation of excised tumors presented in Figure 7C directly revealed that tumor sizes of the PTX/PTS/HQ PNPs group were smaller than those of all other groups. According to Figure 7D, the average weights of isolated tumors in the control, Taxol, PTX/PTS/HA PNPs, and PTX/PTS/HQ PNPs groups were 0.41 g, 0.29 g, 0.25 g, and 0.21 g, respectively, which further confirmed the strongest tumor inhibitory effects of PTX/PTS/HQ PNPs.

#### 3.8.2. Hematoxylin and Eosin (H&E) Staining

An H&E staining assay was utilized to assess the pathological variation in tumor and normal tissues of different treatment groups. The results (Figure 7E) demonstrate that PTX/PTS/HQ PNPs caused the largest areas of tumor necrosis with wrinkled nuclei and cellular vacuolation compared with the other three groups. Furthermore, almost no fibrosis, apoptosis, or inflammatory reaction was detected in major organs after treatment with PTX/PTS/HQ PNPs compared to the control group. These results indicate that PTX/PTS/HQ PNPs can enhance antitumor efficiency and reduce systemic toxicity with good biocompatibility.

#### 3.8.3. Ki67 and TUNEL Assay

Tumor tissue sections were immune-stained with Ki67 and TUNEL antigen to investigate the antiproliferation and apoptosis-inducing capability of PTX/PTS/HQ PNPs, respectively. As shown in Figure 7F (left), the lowest amounts of brown dots were observed in the PTX/PTS/HQ PNPs group among all samples, demonstrating that PTX/PTS/HQ PNPs can remarkably reduce the proliferation of tumor cells. The TUNEL staining image of tumor specimens (Figure 7F (right)) showed that cancer cells exhibited more brown areas with cracked nuclear membranes and chromatin (which represented an increased apoptosis level) after treatment with PTX/PTS/HQ PNPs. These findings were consistent with the in vitro cytotoxicity and apoptosis results in MCF-7/ADR cells (Figure 4C,F,G), further suggesting that the tumor growth inhibition activity of PTX/PTS/HQ PNPs was attributed to the prompted apoptotic effects.

#### 3.8.4. P-Gp Levels Detection

Total proteins were extracted from isolated tumors to determine the expression level of P-gp via the western blot analysis. As presented in Figure 7G, the PTX/PTS/HQ PNPs group inhibited P-gp expression to a larger extent than the other groups, which was similar to the phenomenon described in Figure 5C, implying that PTX/PTS/HQ PNPs can enhance the tumor-suppressive efficiency by decreasing P-gp-mediated chemotherapeutic efflux effects.

#### 3.8.5. Apoptosis-Related Protein Level Detection

As a member of the cysteine aspartic acid protease (Caspase) family, Caspase-3, and its activated state cleaved Caspase-3, proteins play crucial roles in mediating apoptosis [60] and were also evaluated in tumor tissues in this study. According to Figure 7H, the expression level of cleaved Caspase-3 was significantly upregulated after being subjected to the PTX/PTS/HQ PNPs group, suggesting that PTX/PTS/HQ PNPs induce tumor cell apoptosis (Figure 7F) by activating downstream proteins of apoptotic-execution signaling pathways.

**Scheme 2:**
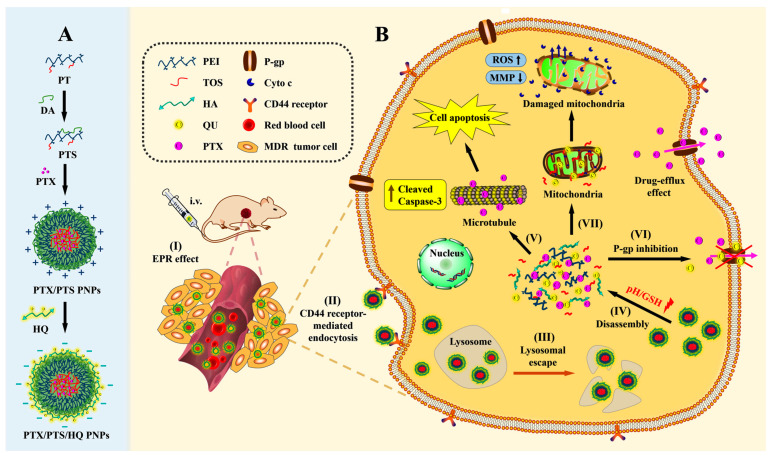
Schematic diagram of self-assembled PTX/PTS/HQ PNPs to conquer MDR tumors. (**A**) The formation process of PTX/PTS/HQ PNPs. (**B**) The in vivo transport pathway and anticancer mechanism of PTX/PTS/HQ PNPs in an MDR tumor-bearing mouse model.

Overall, the pronounced in vitro and in vivo antitumor efficacy of the HA-based multifunctional hybrid PTX/PTS/HQ PNPs can be ascribed to the following aspects, as shown in Scheme 2. After intravenous (i.v.) administration, the PNPs could (I) efficiently accumulate in tumor tissues by EPR effects and then be (II) specifically internalized into tumor cells by means of CD44 receptor-mediated endocytosis. Upon reaching the cytoplasm, the PNPs achieved (III) lysosomal escape through the “proton sponge effect” of PEI. Subsequently, intracellular acid and redox stimulation triggered the cleavage of β-carboxylic amide bonds and -SS-, respectively, leading to (IV) disassembly of the PNPs, followed by the rapid release of QU and PTX to exert synergetic anticancer effects. (V) The chemotherapeutic PTX diffused into microtubules to activate the apoptotic execution protein Caspase-3 and further induced tumor cell apoptosis. Meanwhile, (VI) QU can overcome drug-efflux effects by inhibiting P-gp expression, thereby increasing the intracellular concentration of PTX for improved therapeutic efficacy. Moreover, (VII) TOS and QU can disturb mitochondrial functions by generating excessive ROS, decreasing the MMP, and releasing Cyto c from mitochondria into the cytosol which consequently amplified the chemotherapy efficacy in drug-resistant breast tumors.

## 4. Conclusions

In this study, we successfully designed and prepared an intracellular stimuli-triggered hybrid nanodrug delivery system to simultaneously deliver QU and PTX to tumor sites for multilevel chemotherapy amplification in drug-resistant breast cancer. The obtained self-assembling PTX/PTS/HQ PNPs exhibited a smooth and spherical shape with a particle size of 187.97 ± 1.66 nm, a negative charge of −21.42 ± 0.53 mV, and a favorable mono-dispersity. In addition, PTX/PTS/HQ PNPs had good storage stability and hemocompatibility as well as accelerated drug release ability in acidic/reductive environments. Once entering circulation, PTX/PTS/HQ PNPs can effectively accumulate in tumor sites through EPR effects and subsequently penetrate deep into tumor tissues. Furthermore, they can be selectively internalized by cancer cells via HA/CD44-mediated endocytosis and achieve lysosomal escape with the aid of the “proton sponge effects” of PEI. Moreover, PTX/PTS/HQ PNPs can facilitate intracellular drug enrichment by virtue of the QU-based P-gp efflux inhibition, thus efficiently amplifying the antitumor effect of chemotherapeutic PTX at specific regions. In the MCF-7/ADR tumor-bearing nude mouse model, PTX/PTS/HQ PNPs can substantially suppress tumor growth with an average tumor volume 2.54-fold lower than that of the control group while exhibiting negligible toxicity toward the main organs. The satisfactory antitumor effect of PTX/PTS/HQ PNPs was likely due to the following aspects: (1) the anti-proliferation and proapoptotic effects on tumor cells and (2) the mitochondrial-destruction capability through elevating ROS levels, decreasing the MMP, and promoting the release of Cyto c from mitochondria into the cytosol. In light of these results, we anticipate that the PTX/PTS/HQ PNPs nano-system can provide a valuable reference for the co-delivering of natural compounds and chemotherapeutic agents for satisfactory combination therapy in MDR cancer.

## Figures and Tables

**Table 1:** Characterization of different PNPs, including particle size, PDI, zeta potential, DL (%), and EE (%). Data are presented as the mean values ± SD (*n* = 3).

Samples	Size (nm)	PDI	Zeta (mV)	DL (%)	EE (%)
PT	140.90 ± 0.62	0.20 ± 0.01	43.90 ± 0.56	/	/
PTS	174.70 ± 1.21	0.19 ± 0.02	30.80 ± 0.40	/	/
PTS/HA	151.17 ± 2.25	0.12 ± 0.02	−34.00 ± 0.46	/	/
PTX/PTS	174.70 ± 1.51	0.13 ± 0.05	31.50 ± 0.44	42.85 ± 0.23 ^(a)^	83.43 ± 0.77 ^(c)^
PTX/PTS/HA	179.13 ± 0.81	0.18 ± 0.02	−28.47 ± 0.81	10.67 ± 0.27 ^(a)^	61.83 ± 3.08 ^(c)^
PTX/PTS/HQ	187.97 ± 1.66	0.16 ± 0.02	−21.42 ± 0.53	9.69 ± 0.03 ^(a)^	49.84 ± 0.15 ^(c)^
				1.45 ± 0.12 ^(b)^	

Notes: ^(a)^ represents the DL (%) of PTX; ^(b)^ represents the DL (%) of QU; ^(c)^ represents the EE (%) of PTX.

## Data Availability

Not applicable.

## Supplementary Materials

The following supporting information can be downloaded at: https://www.mdpi.com/article/10.3390/pharmaceutics14020422/s1, Materials, cell culture, and animal model, characterization of PT and PTS copolymers, characterization of HQ conjugates, determination of CMC values, and other relevant methods; Figure S1: The viability of (A) MCF-7 cells, (B) MCF-7/ADR cells, (C) MDA-MB-231 cells, and (D) MDA-MB-231/DOX cells incubated with various concentrations of PTX for 48 h. (E) The summarized table of IC_50_ (μg/mL) and RI values. Data are presented as the mean values ± SD (*n* = 3); Figure S2: The viability of (A) MDA-MB-231/DOX cells and (B) MCF-7/ADR cells incubated with various concentrations of QU for 24 h. Data are presented as the mean values ± SD (*n* = 3); Figure S3: The chemical structure of QU (3,3′,4′,5,7-pentahydroxyflavone); Figure S4: The image of P-gp expression in MDA-MB-231/DOX cells after 24 h incubation. 0, 0.16, 0.24, and 0.32 represent different induced concentrations (μg/mL) of DOX; Figure S5. The typical chromatograms of (A) PTX reference substance solution, (B) PTX/PTS PNPs testing solution, (C) QU reference substance solution, and (D) HQ conjugates testing solution. 1: PTX, 2: QU. Refs. [41,57,61,62] are cited in the Supplementary Materials.
