# Supplementary material for: A Nanosized Codelivery System Based on Intracellular Stimuli-Triggered Dual-Drug Release for Multilevel Chemotherapy Amplification in Drug-Resistant Breast Cancer
Source: Pharmaceutics. 2022 Feb 15;14(2):422. doi: 10.3390/pharmaceutics14020422 (PMC8878749; doi:10.3390/pharmaceutics14020422)
Supplement: Supplementary file 1 [file pharmaceutics-14-00422-s001.zip › pharmaceutics-1579013-supplementary.pdf]

pharmaceutics

# Supplementary Materials: A Nanosized Codelivery System Based on Intracellular Stimuli-Triggered Dual-Drug Release for Multilevel Chemotherapy Amplification in Drug-resistant Breast Cancer

Yufan Guo, Shuo Liu, Fazhen Luo, Dongyun Tang, Tianshu Yang, Xiuru Yang and Yan Xie

## 1. Materials and methods

### *1.1. Materials*

Adipic acid dihydrazide (ADH), dicyclohexylcarbodiimide (DCC), and 2,3-dimethylmaleic anhydride (DMA) were purchased from Aladdin Reagent Inc. (Shanghai, China). Pyrene was purchased from Aladdin Reagent Inc. (Shanghai, China). Ethanol and magnesium sulfate were obtained from Sinopharm Chemical Reagent Co., Ltd. (Shanghai, China). 4′,6-Diamidino-2-phenylindole (DAPI) was bought from Dojindo Molecular Technologies, Inc. (Kumamoto, Japan). Fetal bovine serum (FBS) was obtained from Gibco Laboratory (Grand Island, New York, USA). 3-(4,5-Dimethylthiazol-2-yl)-5-(3-carboxymethoxyphenyl)-2-(4-sulfophenyl)-2H-tetrazolium (MTS) was obtained from Promega Biological Products Co., Ltd. (Shanghai, China). The FITC Annexin V Apoptosis Detection Kit was obtained from BD Biosciences (San Jose, California, USA). The Mitochondrial Membrane Potential Assay Kit with 5,5′,6,6′-tetrachloro-1,1′,3,3′-tetraethylbenzimidazolyl carbocyanine iodide (JC-1), Reactive Oxygen Species Assay Kit, and Cell Mitochondria Isolation Kit were obtained from Beyotime Institute of Biotechnology (Shanghai, China). Bicinchoninic acid (BCA) assay kit was obtained from Beyotime Institute of Biotechnology (Shanghai, China).

### *1.2. Cell culture and animal model*

Human breast cancer cell lines MDA-MB-231, MCF-7, and doxorubicin (DOX)-resistant MCF-7/ADR were purchased from the Institute of Biochemistry and Cell Biology, SIBS, CAS (Shanghai, China). Another DOX-resistant cell lines MDA-MB-231/DOX were induced by our research group through the low-concentration dosage continuous induction method [1]. All cell lines were incubated in DMEM under 37 °C, 5% $CO_2$ atmosphere as well as 10% FBS and 1% antibiotics (100 mg/mL streptomycin plus penicillin) condition. For MDA-MB-231/DOX cells culture, DOX was added in DMEM to achieve the final concentration of 0.32 μg/mL for maintaining the drug-resistant property. In addition, cells grown to 90% confluence were sub-cultured after digested with 0.25% trypsin-EDTA and diluted in fresh growth medium.

Adult Sprague-Dawley (SD) rats (200 ± 20 g) and female BALB/c nude mice (20-25 g) were acquired from Super-B&K Laboratory Animal Corp. Ltd. (Shanghai, China). Food and water are freely available for mice in a specific pathogen-free (SPF) environment at 25 ± 1 °C. After a week of acclimation, these animals were used for experiments. All procedures were approved by the Shanghai University of Traditional Chinese Medicine Laboratory Animal Ethics Committee (Approval number: PZSHUTCM201218005).

MCF-7/ADR human breast tumor models were generated by subcutaneous injection of $8\times10^7$ cells (0.1 mL) in the second right breast region of nude mice. Tumor volumes and body weight of mice were monitored and recorded once every 2 days, and the tumor volume was calculated according to the following formula:

$$V = W^2 \times L/2 \quad (1)$$

where L and W represent the longest and widest diameter of tumor, respectively.

### *1.3. Synthesis of hyaluronic acid-quercetin (HA-QU, HQ) conjugates*

HQ conjugates were synthesized through the indirect conjugation method between hyaluronic acid- adipic acid dihydrazide (HA-ADH) conjugates and DMA-modified quercetin (QU-MA). Briefly, HA (11 kDa, 50 mg) was activated by EDC (48 mg) and NHS (14 mg) in water under pH 4.5, followed by the addition of ADH (436 mg) with gentle stirring. The reaction was terminated by adjusting the pH to 7.0 with sodium hydroxide (NaOH). Then, the mixture solution was dialyzed to remove unbound materials and small molecules using a dialysis bag (3500 Da) against pure water for 48 h, and the final HA-ADH conjugates were obtained by lyophilization (-80 °C, 1 Pa). To obtain QU-MA, QU (30 mg) and DMA (20 mg) were dissolved in ethyl acetate (50 mL) and stirred thoroughly. Then, DCC (24 mg) was added with stirring for 3 days in darkness. The received mixture was concentrated to 3 mL under reduced pressure, followed by the purification through silica gel column chromatography with 2:1 (v/v) petroleum ether/ethyl acetate solution as eluents. Lastly, the resultant bright yellow solid was sealed and stored at 4 °C. The amide reaction was utilized to prepare HQ conjugates. Firstly, QU-MA (30 mg) was activated by EDC (96 mg) and NHS (60 mg) in anhydrous DMSO with gentle stirring for 6 h; followed by the addition of HA-ADH (50 mg) conjugates solution to react for 24 h in darkness. Then, the mixture solution was dialyzed using a dialysis bag (3500 Da) against pure water (pH 7.4) for 48 h, and the final HQ conjugates was obtained by lyophilization (-80 °C, 1 Pa).

### *1.4. Synthesis of polyethyleneimine-tocopherol hydrogen succinate (PEI-TOS, PT) copolymers*

To obtain PT copolymers, tocopherol hydrogen succinate (TOS, 180 mg) was activated by DCC (84 mg) and NHS (46 mg) in anhydrous DMSO with gentle stirring for 3 h; followed by the addition of polyethyleneimine (PEI, 25 kDa, 1 g) solution to react for 24 h. Then, the mixture solution was dialyzed using a dialysis bag (3500 Da) against ethanol and pure water for 24 h and 48 h, respectively. The final PT was obtained by lyophilization (-80 °C, 1 Pa).

### *1.5. Characterization of PT and PTS copolymers, HQ conjugates*

$^1$H-nuclear magnetic resonance ($^1$H NMR) was used to verify the chemical structures of samples with deuterium oxide ($D_2O$) and deuterated methanol ($CD_3OD$) as solvents. The degree of substitution (DS) was calculated using the following equation based on the characteristic peaks:

$$DS\%_{PT} = A_{0.8} / A_{2.5\text{-}3.0} \times 2/3 \times 100\% \quad (2)$$

$$DS\%_{PTS} = A_{3.5\text{-}3.7} / A_{2.5\text{-}3.0} \times 100\% \quad (3)$$

$$DS\%_{HQ} = A_{7.0} / A_{1.9\text{-}2.1} \times 3 \times 100\% \quad (4)$$

where $A_{0.8}$, $A_{2.5\text{-}3.0}$, $A_{3.5\text{-}3.7}$, $A_{7.0}$ and $A_{1.9\text{-}2.1}$ represent the peak areas at 0.8 ppm, 2.5-3.0 ppm, 3.5-3.7 ppm, 7.0 ppm, and 1.9-2.1 ppm in $^1$H NMR spectra of the copolymers and conjugates, which corresponded to the protons of $-CH(CH_3)_2$, $-CH_2-$, $-CH_2-$, 2′H and $-CH_3$ groups in TOS, PEI, DA, QU, and HA, respectively.

### *1.6. Determination of critical micelle concentration (CMC) values*

The CMC values of PTS copolymers were confirmed by the pyrene incorporation method [2]. Briefly, pyrene solution ($6\times10^{-6}$ M) in acetone was prepared in advance, subsequently adding the prepared copolymer solutions with different concentrations (0.75-3.5 mg/mL). After incubation in darkness overnight at 25 °C, the fluorescence emission spectra of pyrene were recorded from 300 to 500 nm using a fluorescence spectrophotometer (F-4500, Hitachi, Japan) with an excitation wavelength of 338 nm. Finally, the intensity ratios between the emission spectrum at 374 nm and 384 nm ($I_{374}/I_{384}$) were plotted against the micelle concentration to determine the CMC.

### *1.7. Preparation and characterization of PT, PTS, and PTS/HA blank PNPs*

To obtain PT and PTS blank PNPs, PT or PTS copolymers (DS%=19.8%, 10 mg) were dissolved in pure water (10 mL) and stirred thoroughly, followed by the sonication (10 min, 100 W) using a probe-type sonicator in an ice bath. Next, PTS/HA blank PNPs were received after the addition of HA solution (1 mg/mL) in PTS blank PNPs with stirring for

5 min. Particle size distribution, polydispersity index (PDI), zeta potential, and morphology of each sample were ultimately determined by dynamic light scattering (DLS) and transmission electron microscopy (TEM), respectively.

*1.8. Drug entrapment efficiency and loading capacity*

The concentration of paclitaxel (PTX) and quercetin (QU) were examined using high-performance liquid chromatography (HPLC, Agilent 1260 system, California, USA) equipped with a Kromasil 100-5C18 column (250 mm × 4.6 mm, 5 μm) at a temperature of 37 °C. To measure the PTX content, PTX/PTS PNPs and PTX/PTS/HQ PNPs were destroyed by methanol with sonication for 15 min to obtain free PTX. Besides, the 55:45 (v/v) acetonitrile/water solution was used as the mobile phase and 227 nm as the detection wavelength. To measure the QU content, acetic acid was applied to break off the pH-sensitive bond in HQ, and the chromatographic conditions were as follows: mobile phase, 50:50 (v/v) methanol/0.2% phosphoric acid water; detection wavelength, 371 nm. Additionally, the mobile phase was delivered at a flow rate of 1 mL/min for both PTX and QU, the injected volume of each sample was 20 μL. Drug loading (DL) and encapsulation efficiency (EE) rate were calculated by the following equations.

$$DL\%_{QU} = \text{amount of QU in HQ/ total amount of HQ} \times 100\% \quad (5)$$

$$DL\%_{PTX} = \text{amount of PTX in PMs/ total amount of PMs} \times 100\% \quad (6)$$

$$EE\%_{PTX} = \text{amount of PTX in PMs/ initial amount of PTX added} \times 100\% \quad (7)$$

*1.9. Responsive behaviors of PTX/PTS PNPs and HQ conjugates*

To investigate the reduction sensitivity, PTX/PTS PNPs were dispersed in PBS with or without 40 mM GSH under gentle stirring. At given time intervals, the changes in particle size were observed by DLS. The *in vitro* GSH induced PTX-release of PTX/PTS PNPs were also conducted by sediment method as described in the Main Text 2.4. Similarly, two different PBS solution (pH 7.5 or pH 6.5) were utilized to investigate the acid-triggered QU-release of HQ conjugates through dialysis method as described in the Main Text 2.4.

*1.10. Storage stability*

To detect the stability under different storage conditions (4 °C and 25 °C), the particle size of PTS blank PNPs and PTS/HA blank PNPs were determined by DLS at predetermined time points (0, 1, 2, 4, 8, 15, 20, 25, and 30 days). Similarly, after incubated for 0, 1, 3, 7, and 10 days, the size and zeta potential of PTX/PTS/HQ PNPs were monitored by DLS analysis. In addition, the concentrations of PTX were also determined by HPLC described in Supplementary Materials 1.8. at specific time intervals for 10 days.

*1.11. Hemolysis test*

Hemolytic activities of PTS blank PNPs, PTS/HA blank PNPs, PTX/PTS/HA PNPs, and PTX/PTS/HQ PNPs were assessed by monitoring the release of hemoglobin from rat blood cells. Firstly, fresh blood was taken from the eye socket of SD rats, the red blood cells (RBCs) were collected by centrifuging (3500 rpm, 10 min) and washed multiple times using saline solution to remove serum. After being diluted, 0.5 mL 2% RBCs suspension was incubated with equal volume of above four formulations (equivalent to 0.1-2 mg/mL of PTS) at 37 °C with gentle shaking for 1 h. Besides, RBCs suspension only treated with pure water and saline under the same condition were used as positive (100% hemolysis) and negative (0% hemolysis) control, respectively. At 1 h post incubation, the reaction solution was transferred for centrifugation (3500 rpm, 10 min) to remove RBCs, and the released hemoglobin out of RBCs in the supernatant was quantified based on absorbance measurement at 540 nm with a microplate reader (Spectra Max iD5, Molecular Devices, USA). Finally, the hemolysis rate was defined using the following equation:

$$\text{Hemolysis rate (\%)} = (A_{sam} - A_{neg}) / (A_{pos} - A_{neg}) \times 100\% \quad (8)$$

where $A_{sam}$, $A_{neg}$, and $A_{pos}$ represent the absorbance of the sample, negative control, and positive control, respectively.

*1.12. In vitro cytotoxicity test*

The MTS assay was utilized to evaluate the anti-proliferative activity. Firstly, MCF-7 cells, MCF-7/ADR cells, MDA-MB-231 cells, and MDA-MB-231/DOX cells were incubated in 96-well plates ($1\times10^4$ cells/well) with 100 μL DMEM overnight, respectively. Then, the growth medium was replaced by 100 μL fresh medium containing various concentrations of PTX and incubated for 48 h to confirm the PTX-resistant property of cancer cells. Besides, various concentrations of QU (1, 10, 20, 30, 40, 50, 60 μg/mL) were exposed to MCF-7/ADR cells for 24 h to detect the cytotoxicity. As for the synergetic cytotoxicity effects of PTX and QU, QU solution (10, 30, and 60 μg/mL) was added to MCF-7/ADR cells in advance and incubated overnight, after that, cells were further treated with PTX solution ranging from 0.005 to 0.7 μg/mL for 48 h. For the biocompatibility evaluation, the medium of MCF-7/ADR cells and MDA-MB-231/DOX cells were removed and replaced with 100 μL fresh medium containing PTS or PTS/HA blank PNPs (equal to 0.02-70 μg/mL PTS) after cells adhered and then treatment for 48 h. To study the *in vitro* antitumor therapeutic potential of drug-loaded PMs, the growth medium of MCF-7/ADR cells was replaced by 100 μL fresh medium containing PTX suspension, PTX/PTS/HA PNPs, or PTX/PTS/HQ PNPs in equivalent to PTX concentration ranging from 0 to 10 μg/mL and further cultured for 48 h.

Finally, the medium was removed followed by the addition of MTS solution and continuously incubated for another 45 min, then the absorbance of each well at 490 nm was determined with a microplate reader. The cell availability was calculated by the following equation:

$$\text{Cell viability (\%)} = (A_{sample} - A_{blank}) / (A_{control} - A_{blank}) \times 100\% \quad (9)$$

where 0.5% DMSO or 1% pure water was used as the control, and the half maximal inhibitory concentrations ($IC_{50}$) values were calculated through GraphPad Prism 6.0. Then the resistance index (RI) and RF values were calculated using the following formula:

$$RI = IC_{50\ resistant\ cells} / IC_{50\ sensitive\ cells} \quad (10)$$

$$RF = IC_{50\ the\ PTX\ alone} / IC_{50\ the\ combination\ of\ PTX\ and\ QU} \quad (11)$$

### *1.13. Cell apoptosis assay*

Firstly, $8\times10^5$ MCF-7/ADR cells were seeded in 6-well plates and incubated overnight for attachment. To detect the synergetic effect of PTX and QU, QU solution (5, 10, and 20 μg/mL) was added in advance and incubated overnight, then cells were further treated with PTX solution (0.3 μg/mL) for 48 h. For studying the *in vitro* therapeutic potential of drug-loaded PNPs, PTX suspension, PTX/PTS/HA PNPs, and PTX/PTS/HQ PNPs with PTX concentration of 10 μg/mL were exposed to cells for 48 h. Afterwards, cells were harvested and washed with binding buffer, subsequently were incubated with annexin V-FITC and then with propidium iodide (PI) for 15 min in darkness. Finally, fluorescence images were obtained through flow cytometry (FACS Calibur, BD, USA) within 1 hour.

### *1.14. Construction of three-dimensional (3D) tumor spheroids*

MCF-7/ADR breast cancer tumor spheroids were prepared using liquid overlay method [3]. Briefly, 24-well plates were coated with autoclaved agarose (1%, 300 μL per well), MCF-7/ADR cells were then seeded at a density of $6\times10^4$ cells/well and centrifuged for 1 min at 1000 g. These plates were then kept under incubation at 37 °C and 5% $CO_2$ until its size reached 600 ± 10 μm. The formation of spheroids was monitored by using a microscope, and fresh media was replaced on alternative days. Compact and uniform tumor spheroids were selected for further use in 3D tumor spheroids penetration experiments.

### *1.15. In vitro endosomal escape analysis*

Briefly, MDA-MB-231/DOX cells ($1\times10^5$ cells/well) were seeded in glass bottom dishes and cultured overnight for attachment. The growth medium was further removed and cells were incubated with coumarin-6 ($C_6$)/PTS/HQ PNPs, in which the final concentration of $C_6$ was 0.1 μg/mL. At predetermined time points (2, 4, and 6 h), cells were washed with cold PBS and fixed with 4% (w/v) paraformaldehyde. Then lysosomes and

nuclei were stained with 1 mL Lysotracker Green for 30 min and 1 mL DAPI for 20 min in darkness as per manufacturer's instruction, respectively. Thereafter, CLSM was utilized to obtain cell fluorescence images with an excitation/emission wavelengths of 360/454 nm for DAPI, 504/511 nm for Lysotracker Green, and 466/504 nm for $C_6$, respectively.

*1.16. In vitro HA-targeted cellular internalization*

To investigate the CD44 receptor-mediated endocytosis of $C_6$/PTS/HQ PNPs, HA competitive inhibition experiments were performed as follows. Firstly, MDA-MB-231/DOX cells were seeded in glass bottom plates at a density of $4\times10^5$ cells per well with 24 h adherent growth. Then the medium was sucked out and replaced with 2 mL fresh DMEM containing free $C_6$ and $C_6$/PTS/HQ PNPs (equivalent to 0.1 μg/mL $C_6$) for 4 h incubation. As for the HA-inhibited group, cells were co-cultured with free HA (10 mg/mL) for 2 h in advance before adding $C_6$/PTS/HQ PNPs for another 4 h treatment. Following procedures were carried out similarly to the cellular uptake study as described in the Main Text 2.5.

*1.17. Western blot*

For P-glycoprotein (P-gp) expression, MDA-MB-231/DOX cells were planted in 6-well plates ($4\times10^5$ cells per well) overnight. Further, DMEM was removed and cells were exposed to free QU (5.4 μg/mL), PTS/HA blank PNPs, and PTS/HQ blank PNPs (equal to 20 μg/mL PTS) for 24 h incubation. After sufficient PBS washing, cells in all groups were harvested, and then lysed with RIPA buffer (containing protease and phosphatase inhibitor cocktails) on the ice for 30 min. Subsequently, cell lysates were centrifuged (1000 g, 4 °C) for 10 min followed by the BCA assay to quantify the total protein concentration. Equal amounts of protein were separated by sodium dodecyl sulfate polyacrylamide gel electrophoresis (SDS-PAGE, 5% stacking gel and 12% separating gel) and transferred onto polyvinylidene difluoride (PVDF) membranes. After immersed in blocking buffer (20 min), the membrane was incubated with primary antibody at 4 °C overnight and then incubated with secondary antibody at 37 °C for 2 h. Protein bands were finally visualized using Chemiluminescent Imaging System (ESCO, Singapore) and the presented quantitative results were measured with ImageJ software. β-actin was used as internal control.

*1.18. Morphological assay of mitochondria*

MCF-7/ADR cells were grown in glass-bottom dishes at a density of $4\times10^5$ cells per well overnight for attachment. Then the growth medium was replaced with PTS/HA and PTS/HQ blank PNPs (equal to 20 μg/mL PTS) for 24 h incubation. Cells were then washed twice with PBS and cultured with Mito-Tracker Green (200 nM) for 45 min in darkness at 37 °C. Finally, images of mitochondria were acquired using CLSM with excitation and emission wavelengths set at 490 nm and 516 nm, respectively.

*1.19. Detection of reactive oxygen species (ROS) levels*

The production of intracellular ROS was determined using DCFH-DA (a free radical probe). Firstly, MCF-7/ADR cells were seeded into 6-well plates ($4\times10^5$ cells/well) and cultured overnight, followed by exposure to Rosup (positive control), free QU, PTS/HA, and PTS/HQ blank PNPs (equal to 20 μg/mL PTS), respectively. After another 24 h treatment, cells were collected, suspended, and incubated with DCFH-DA (1 mL) for 1 h in darkness according to the manufacturer's protocol. Finally, cells were washed and resuspended by PBS, and the flow cytometry was employed to measure the fluorescence intensity immediately.

*1.20. Mitochondrial membrane potential (MMP) determination*

The MMP was measured using JC-1 dye (a fluorescent probe) to detect the color variation of treated cells. Briefly, MCF-7/ADR cells were seeded in glass-bottom dishes with a density of $1\times10^4$ cells/well. After incubation overnight for proper cell attachment, DMEM was replaced with PTS/HA and PTS/HQ blank PNPs (equal to 20 μg/mL PTS) for 24 h treatment. Then, cells were stained with 2 mL JC-1 solution according to the manufacturer's protocol for 40 min at 37 °C. Finally, fluorescence images were captured by CLSM

with Ex (490 nm)/ Em (530 nm) for JC-1 monomers (green) and Ex (525 nm)/ Em (590 nm) for JC-1 aggregates (red), respectively.

*1.21. Cytochrome c (Cyto c) release*

The expression level of Cyto c in MCF-7/ADR cells was measured through the western blot analysis. Briefly, cells were seeded in culture dishes with a density of $3 \times 10^6$ cells/well overnight for attachment. Then the growth medium was replaced with PTS/HA and PTS/HQ blank PNPs (equal to 20 μg/mL PTS) for 24 h incubation. Cells were subsequently digested, harvested, and washed thrice with ice-cold PBS. Thereafter, mitochondrial and cytosolic protein fractions were isolated using the Cell Mitochondria Isolation kit according to instructions. Following standard procedures were performed as described in Supplementary Materials 1.17.

*1.22. Histological analysis*

After mice sacrificed, tumors and main organs were collected and fixed with 4% paraformaldehyde for 24 h. Subsequently, samples were embedded in paraffin and sliced into 5 μm-thick sections. Isolated tumors were stained with hematoxylin and eosin (H&E), Ki67 kits (Service-bio, China), and TUNEL apoptosis assay kit (Roche, China) to assess histological alterations using an optical microscope. Other organs were only performed with H&E staining for biosafety analysis.

*1.23. Detection of P-gp, Caspase-3, and cleaved Caspase-3 protein levels in tumor tissues*

After the sacrifice of mice, dissected tumor tissues were further used for western blot assay. Briefly, tumors were cut into pieces and washed with saline. After being triturated on ice bath, samples were adequately homogenized in lysis buffer for 1 h. Subsequently, the lysates were centrifuged (13000 rpm) at 4 °C for 5 min. Following standard procedures were performed as described in Supplementary Materials 1.17.

## 2. Results

*2.1. The sensibility of breast cancer cell lines to PTX*

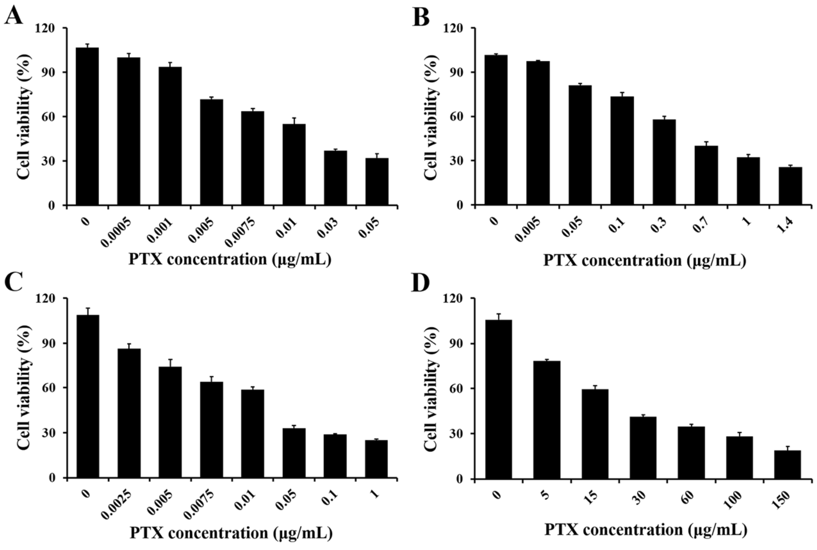


E

| Cells | $IC_{50}$ | RI |
|---|---|---|
| MCF-7 | 0.016 | 1.0 |
| MCF-7/ADR | 0.398 | 24.9 |
| MDA-MB-231 | 0.026 | 1.0 |
| MDA-MB-231/DOX | 23.787 | 904.9 |

**Figure S1.** The viability of (**A**) MCF-7 cells, (**B**) MCF-7/ADR cells, (**C**) MDA-MB-231 cells, and (**D**) MDA-MB-231/DOX cells incubated with various concentrations of PTX for 48 h. (**E**) The summarized table of $IC_{50}$ (μg/mL) and RI values. Data are presented as the mean values ± SD ($n = 3$).

It reported that MCF-7/ADR cell lines were a typical DOX-resistant human breast cancer cell lines with cross resistance to PTX, which have been widely used in MDR tumor study [4]. Another DOX-resistant cell lines were established by treating MDA-MB-231 cells with gradually increasing concentrations of DOX through a period of 6 months. As shown in Figure S1A-S1D, the viability of MCF-7, MCF-7/ADR, MDA-MB-231, and MDA-MB-231/DOX cell lines gradually decreased with the growing concentrations of PTX, and the $IC_{50}$ values shown in Figure S1E are 0.016 μg/mL, 0.398 μg/mL, 0.026 μg/mL, and 23.787 μg/mL, respectively. In addition, the RI values of MCF-7/ADR cells and MDA-MB-231/DOX cells were 24.9 and 904.9, respectively, which suggest that they are resistant to PTX and therefore can be used in the following MDR reversal experiments.

*2.2. The cytotoxicity of QU*

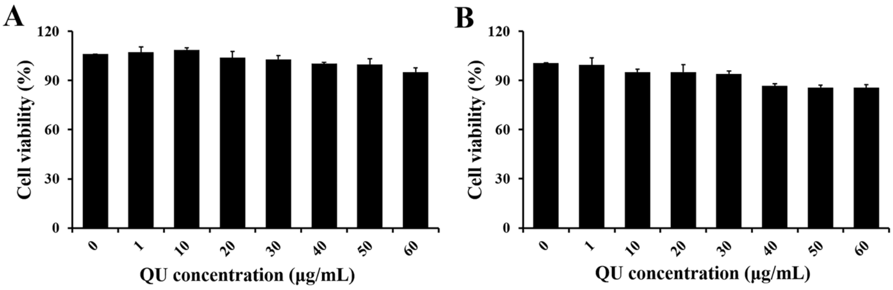


**Figure S2.** The viability of (**A**) MDA-MB-231/DOX cells and (**B**) MCF-7/ADR cells incubated with various concentrations of QU for 24 h. Data are presented as the mean values ± SD ($n$ = 3).

The cytotoxicity of QU with the concentration from 0 to 60 μg/mL was studied and the results are revealed in Figure S2. It is found that QU showed little cytotoxicity on both cell lines at the presupposed concentration range with the survival rates nearly of 90%. Therefore, the concentration of 10, 30, and 60 μg/mL QU were used for the following experiments to test the synergetic anticancer effects of PTX and QU.

*2.3. The chemical structure of QU*

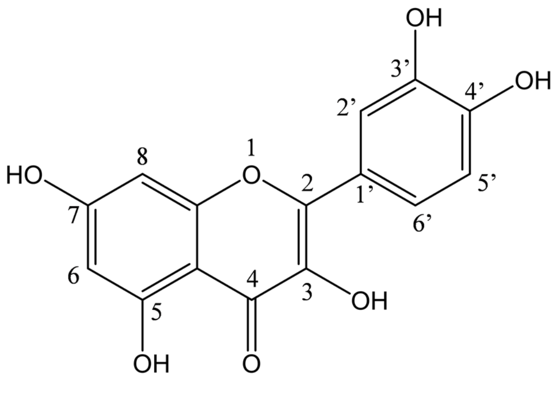


**Figure S3.** The chemical structure of QU (3,3′,4′,5,7-pentahydroxyflavone).

*2.4. The protein expression of P-gp in MDA-MB-231/DOX cell lines*

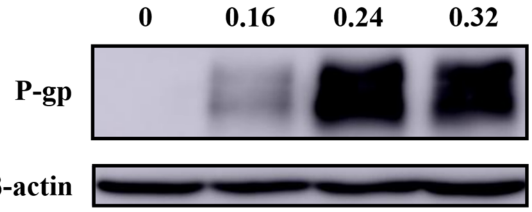


**Figure S4.** The image of P-gp expression in MDA-MB-231/DOX cells after 24 h incubation. 0, 0.16, 0.24, and 0.32 represent different induced-concentrations (μg/mL) of DOX.

*2.5. The HPLC analysis of PTX and QU*

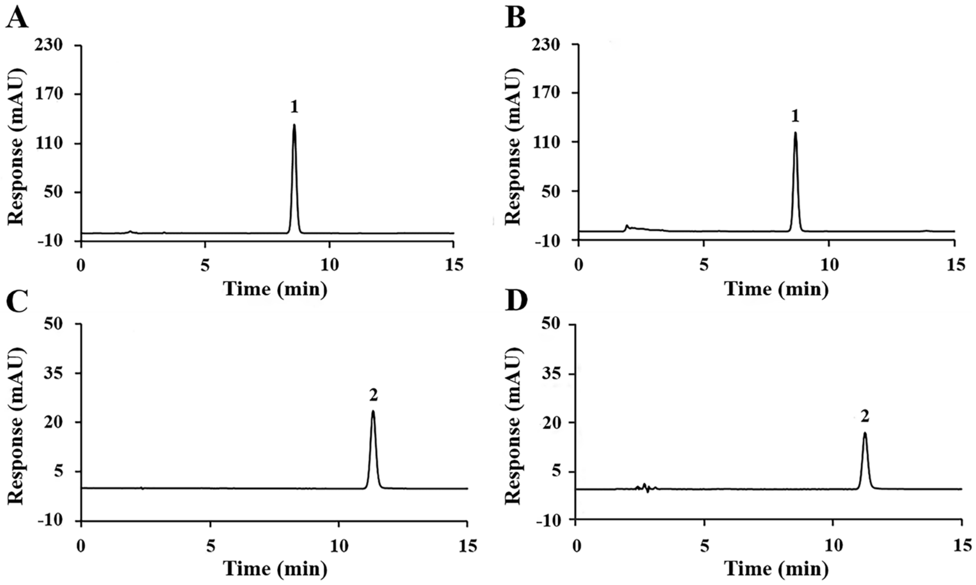


**Figure S5.** The typical chromatograms of (**A**) PTX reference substance solution, (**B**) PTX/PTS PNPs testing solution, (**C**) QU reference substance solution, and (**D**) HQ conjugates testing solution. 1: PTX, 2: QU.

The standard curve method was employed to analyze the concentration of PTX and QU. As shown in Figure S5, all samples exhibited a symmetrical peak shape and excellent separation without any interference. The retention time was about 8.7 minutes (Figure S5B, PTX) and 11.8 minutes (Figure S5D, QU) of the PTX/PTS PNPs testing solution and HQ conjugates testing solution, respectively; which coped well with the retention time of PTX reference substance solution (Figure S5A) and QU reference substance solution (Figure S5C), respectively. These results indicated that the chromatographic conditions described in Supplementary Materials 1.8. can be used for the quantitative determination of PTX and QU, respectively.

## References

1. Han, M.; Vakili, M.R.; Soleymani Abyaneh, H.; Molavi, O.; Lai, R.; Lavasanifar, A. Mitochondrial delivery of doxorubicin via triphenylphosphine modification for overcoming drug resistance in MDA-MB-435/DOX cells. *Mol. Pharm.* **2014**, *11*, 2640–2649.
2. Zhang, Y.; Cui, Z.; Mei, H.; Xu, J.; Zhou, T.; Cheng, F.; Wang, K. Angelica sinensis polysaccharide nanoparticles as a targeted drug delivery system for enhanced therapy of liver cancer. *Carbohydr. Polym.* **2019**, *219*, 143–154.
3. Lamichhane, S.P.; Arya, N.; Kohler, E.; Xiang, S.; Christensen, J.; Shastri, V.P. Recapitulating epithelial tumor microenvironment in vitro using three-dimensional tri-culture of human epithelial, endothelial, and mesenchymal cells. *BMC Cancer* **2016**, *16*, 581.
4. Shi, X.; Yang, X.; Liu, M.; Wang, R.; Qiu, N.; Liu, Y.; Yang, H.; Ji, J.; Zhai, G. Chondroitin sulfate-based nanoparticles for enhanced chemo-photodynamic therapy overcoming multidrug resistance and lung metastasis of breast cancer. *Carbohydr. Polym.* **2021**, *254*, 117459.
